# Associations between *LPL* gene polymorphisms and coronary artery disease: evidence based on an updated and cumulative meta-analysis

**DOI:** 10.1042/BSR20171642

**Published:** 2018-03-16

**Authors:** Wen-Qi Ma, Ying Wang, Xi-Qiong Han, Yi Zhu, Nai-Feng Liu

**Affiliations:** Department of Cardiology, Zhongda Hospital, School of Medicine, Southeast University 87 Dingjiaqiao, Nanjing 210009, P.R. China

**Keywords:** coronary artery disease, gene polymorphisms, lipoprotein lipase, meta-analysis

## Abstract

Lipoprotein lipase (LPL) is widely linked to lipid and lipoprotein metabolism, but its effects on coronary artery disease (CAD) are not clearly elucidated. The aim of the present study was to clarify the association between *LPL* gene polymorphisms and CAD susceptibility. The pooled odds ratio (OR) and 95% confidence interval (CI) were calculated to estimate the strength of the relationship between *LPL* gene polymorphisms and CAD risk. Comprehensive electronic databases, including PubMed, EMBASE, Web of Science, and the Cochrane Library, were systematically searched. A total of 45 records containing 80 eligible studies were analyzed. The results indicated an increased risk between the *LPL* D9N polymorphism and susceptibility to CAD in the dominant genetic model (AA + GA vs. GG: OR = 1.46, 95% CI = 1.14–1.87), whereas the *LPL* HindIII polymorphism showed a protective effect against CAD under all tested models (GG + GT vs. TT: OR = 0.85, 95% CI = 0.75–0.97; GG vs. TT + TG: OR = 0.62, 95% CI = 0.47–0.83; G vs. T: OR = 0.81, 95% CI = 0.71–0.92). No significant association was identified for the *LPL* N291S and PvuII polymorphisms. Stratification analysis by ethnicity suggested a significant correlation between the *LPL* S447X polymorphism and CAD susceptibility in Caucasians under the dominant and allele genetic models. In summary, our meta-analysis indicated that the *LPL* D9N polymorphism was associated with an increased risk of CAD, whereas the S447X and HindIII polymorphisms showed protective effects. There was no association observed between the N291S and PvuII polymorphisms and CAD risk.

## Introduction

Coronary artery disease (CAD) is a complex multifactorial disease and a leading cause of morbidity and mortality worldwide [1]. Although genetic and environmental factors have been widely implicated in the mechanisms underlying the pathogenesis of CAD, these potential factors remain an area of active investigation [2]. Atherosclerosis is the underlying cause of CAD, which is primarily characterized by excessive lipid deposition in the endothelium of the vascular tree walls [3]. Individuals with aberrant lipid and lipoprotein metabolism, including elevated levels of triglyceride (TG), cholesterol (TC), and low-density lipoprotein cholesterol (LDL-C) and decreased levels of high-density lipoprotein cholesterol (HDL-C), are more inclined to the development of CAD [4]. Genome-wide association studies (GWAS) have also identified nearly 150 loci linked to plasma lipid traits, and some of these loci are associated with altered lipoprotein lipase (LPL) gene expression [5]. Furthermore, several studies have demonstrated a causal link between triglyceride-rich lipoproteins (TRLs) and CAD, with variants in several crucial genes involved in TRLs metabolism, including LPL and its regulators [2,6]. In the past decades, numerous studies have reported that the *LPL* gene variants directly affect abnormal lipid and lipoprotein metabolism and its influence on the risk of CAD [7–9]. However, the underlying mechanisms that mediate these effects remain poorly elucidated.

LPL is a glycoprotein containing 448 amino acids, which is synthesized and secreted by various tissues, such as adipose tissue, myocardium, and skeletal muscle [10]. As an important component in TRL metabolism, LPL binds to the capillary endothelium and primarily hydrolyzes TGs in circulating TRLs, chylomicrons (CM), and very-low-density lipoproteins (VLDL), providing fatty acids for the energy requirements of the heart and skeletal muscle and for storage [5,10].

The *LPL* gene maps to chromosome 8p22, and over 100 various mutations have been identified [11,12]. Several genetic variants in the *LPL* gene have been reported to be associated with CAD susceptibility [13–15]. However, the results were conflicted, and no general agreements existed between them. For example, the D9N (rs1801177, G to A mutations) and N291S (rs268, A to G mutations) polymorphisms, which both result in partial defects in LPL catalytic function, are reported to be associated with an increased risk of CAD [16–18]. Similarly, the HindIII (rs320, T to G mutations) and PvuII (rs285, C to T mutations) variant sites (located on introns 8 and 6 respectively), which are related to profound alterations in plasma lipids, also seemed to be associated with CAD [9,14]. However, other studies did not confirm these results [19–21]. Meanwhile, several gain-of-function *LPL* variants, such as the S447X (rs328) polymorphism, which lead to the transition of Serine (S) to a stop codon (X) at codon position 447, result in reduced TG levels and an overall favorable lipid profile [5]. In addition, certain studies demonstrated that carriers of the X447 allele are protected against CAD [22–24], while other studies drew the opposite conclusion [13,25].

To confirm the correlation existed between the *LPL* gene polymorphisms (HindIII, S447X, N291S, D9N, and PvuII) and CAD, we performed this meta-analysis by pooling all eligible studies to calculate the estimate of overall CAD risk.

## Methods and materials

### Literature search strategy

We performed the present study according to the MOOSE (Meta-analysis of Observational Studies in Epidemiology) guidelines for meta-analysis of observational studies (Supplementary Table S1) [26]. The literature search was performed by two authors (W.-Q.M. and Y.Z.). PubMed, Web of Science, EMBASE, and the Cochrane Library were systematically searched, and the time period for references searching was from the first available article to September 2017. The following search terms were applied: (“lipoprotein lipase” or “LPL” or “N291S” or “S477X” or “D9N” or “HindIII” or “PvuII”) and (“genetic polymorphisms” or “mutation” or “variant” or “polymorphism”) and (“coronary artery disease” or “coronary heart disease” or “atherosclerosis” or “acute coronary syndrome” or “angina” or “myocardial infarction”). Handsearching was also carried out to find potential relevant records.

### Inclusion and exclusion criteria

The following criteria were applied for reference selection: (1) studies on the evaluation of the *LPL* gene polymorphisms (HindIII, S477X, D9N, N291S, and PvuII) and CAD susceptibility; (2) total CAD cases were documented by angiographic evidence of at least 50% stenosis of one major coronary vessel, myocardial infarction, angina, a history of prior angioplasty, or coronary artery bypass surgery; (3) the data in the reference were sufficient for the present estimation, such as the total number of cases and controls, distribution of genotypes or other relevant information; and (4) the language was limited to English. Studies were excluded if they met any of the following criteria: (1) non-English record; (2) abstracts, letters to the editor, reviews, case-only studies, meta-analysis, and animal studies; and (3) study with useless or insufficient data and multiple publications that reported the same or overlapping population information.

### Data extraction

Data abstraction was independently performed by two investigators (W.-Q.M. and X.-Q.H.), and disagreements about study selection were discussed and resolved by a third investigator (N.-F.L.). The following information was extracted from each included article: author, publication date, ethnicity, total number of cases and controls, country, sources of controls, genotyping methods, genotype frequency in cases and controls, and Hardy–Weinberg equilibrium (HWE) in the controls.

### Quality assessment

The Newcastle–Ottawa scale (NOS) was applied in the quality assessment [27]. The validated quality assessment instrument was composed of the following three parameters of quality: selection, comparability, and exposure assessment. NOS scores ranged from zero to nine. Studies with an NOS score of five or greater were considered moderate to high quality studies, whereas those with an NOS score of less than five were considered low quality.

### Statistics analysis

Pooled odds ratios (ORs) and 95% confidence intervals (CIs) were applied to estimate the strength of association between the *LPL* gene polymorphisms and CAD susceptibility. The dominant, recessive, and allele genetic models were applied to assess the correlation between the *LPL* HindIII, PvuII, and S477X gene polymorphisms and CAD risk. Only the dominant genetic model was applied for N291S and D9N, due to the low number of minor homozygotes. The Cochrane *Q*-test and index (*I*^2^) were calculated to evaluate the heterogeneity within studies. *P*-value < 0.1 in the *Q*-test or *I*^2^ > 50% indicated significant heterogeneity. According to the strength of heterogeneity among studies, the fixed- or random-effects model was applied to calculate the OR and the corresponding 95% CI. The *Z*-test was used to determine the significance of overall ORs. Subgroup analyses, which were based on ethnicity (Asians and Caucasians) and sample size (studies with more than 500 subjects were categorized as “large,” and studies with less 500 subjects were categorized as “small”), were applied to detect sources of heterogeneity. In addition, the influence of sample sizes on the overall risk estimation was assessed by a cumulative meta-analysis [28]. A sensitivity analysis was performed to assess the stability of the individual studies. Possible publication bias was assessed using funnel plots and the Egger linear regression test. All calculations were performed and graphs were made with Review Manager v5.2 (The Cochrane Collaboration, Oxford, U.K.) and Stata 12.0 (Stata Corporation, College Station, Texas, U.S.A.).

## Results

### Selection and characteristics of studies

**Figure 1:**
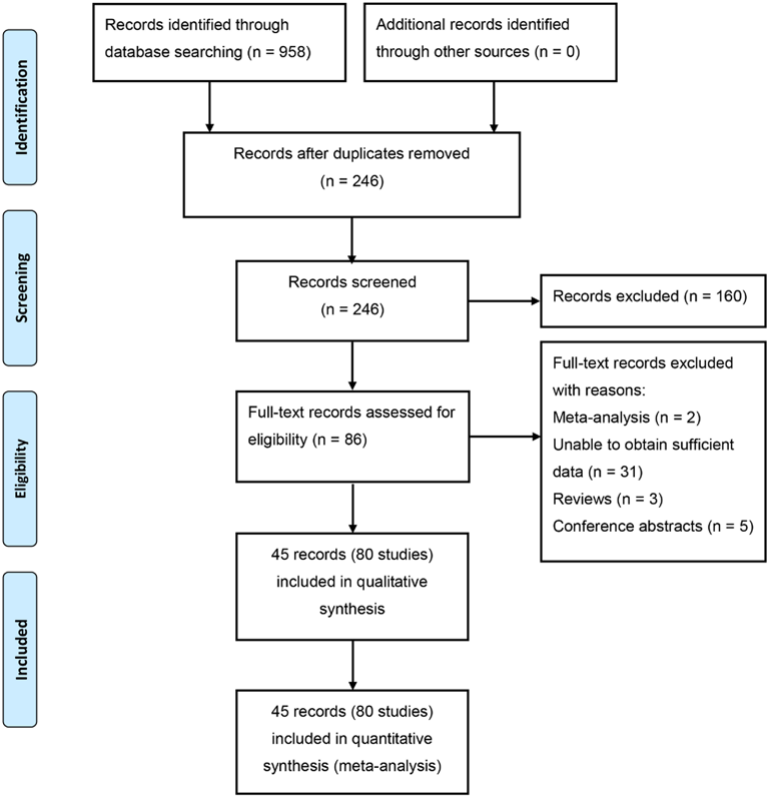
Flow diagram of the study selection process

A total of 958 articles were acquired after initial searching. Among them, 712 duplicate articles were excluded, and 160 articles were excluded for ineligibility after screening the titles and abstracts. In addition, 41 articles were excluded because of insufficient data, reviews, meta-analyses, or conference abstracts. Finally, 45 articles containing 80 eligible studies were included in this meta-analysis [7–9,13–25,29–57]. The flow chart of the retrieved and excluded studies with specifications of reasons is summarized in Figure 1.

The characteristics of the studies included in the meta-analysis are shown in Supplementary Table S2. Among 80 eligible studies, 18 studies, containing 5532 cases and 4813 controls, correlated the *LPL* HindIII polymorphism with susceptibility to CAD. Twenty-seven studies, involving 6959 cases and 9400 controls, focused on the relationship between the *LPL* S447X polymorphism and susceptibility to CAD. Eleven studies, including 9272 cases and 15,074 controls, focused on the relationship between the *LPL* N291S polymorphism and susceptibility to CAD. Eight studies, involving 2583 cases and 2525 controls, focused on the relationship between the *LPL* D9N polymorphism and susceptibility to CAD, and the remaining 16 studies, involving 7831 cases and 5966 controls, concerned the *LPL* PvuII polymorphism. The countries in which these studies occurred included the U.S.A., U.K., France, Brazil, China, Finland, and others. HWE had been applied for all polymorphisms in the controls. The quality of these enrolled studies was evaluated using the NOS quality scale (Supplementary Table S3).

### Association between the *LPL* HindIII polymorphism and susceptibility to CAD

**Figure 2:**
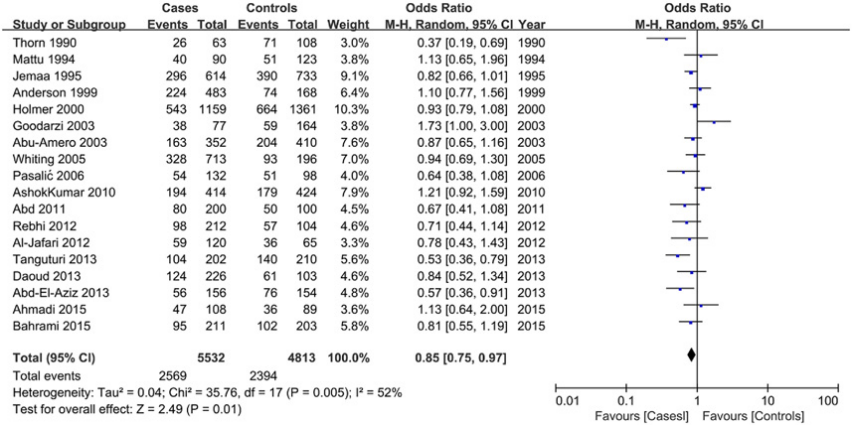
Forest plot of odds ratios for the association between the *LPL* HindIII polymorphism and CAD risk under dominant genetic model (GG + GT vs. TT)

In all study subjects, the results indicated a reduced risk of CAD susceptibility associated with the *LPL* HindIII polymorphism in all tested genetic models (GG + GT vs. TT: OR = 0.85, 95% CI = 0.75–0.97; GG vs. TT + TG: OR = 0.62, 95% CI = 0.47–0.83; G vs. T: OR = 0.81, 95% CI = 0.71–0.92) with some evidence of interstudy heterogeneity (Table 1; Figure 2). Stratification analysis by ethnicity and sample size indicated a significant association between the HindIII polymorphism and CAD susceptibility in Caucasians and small sample size under all tested models (Table 1; Supplementary Figures S1 and S2).

**Table 1:** Summary of odds ratios (95% CI) in the analysis of the association between the *LPL* HindIII polymorphism and CAD susceptibility

Genetic model	Overall and subgroups	*N*	Test of association			Test of heterogeneity	
			OR	95% CI	*P*-value	*P*_Heterogeneity_	*I*^2^ (%)
**GG + GT vs. TT**	Overall	18	0.85	0.75,0.97	0.010	0.005	52%
	Asians	7	0.86	0.70,1.07	0.190	0.050	52%
	Caucasians	9	0.81	0.69,0.96	0.010	0.040	51%
	Large sample	6	0.94	0.85,1.05	0.300	0.320	15%
	Small sample	12	0.76	0.62,0.94	0.010	0.020	53%
**GG vs. TT + TG**	Overall	18	0.62	0.47,0.83	0.001	0.000	67%
	Asians	7	0.67	0.43,1.06	0.090	0.004	69%
	Caucasians	9	0.58	0.38,0.88	0.010	0.000	71%
	Large sample	6	0.82	0.60,1.12	0.220	0.030	60%
	Small sample	12	0.50	0.34,0.75	0.000	0.006	58%
**G vs. T**	Overall	18	0.81	0.71,0.92	0.001	0.000	72%
	Asians	7	0.82	0.65,1.05	0.110	0.000	77%
	Caucasians	9	0.78	0.66,0.92	0.003	0.001	69%
	Large sample	6	0.94	0.85,1.04	0.200	0.180	35%
	Small sample	12	0.73	0.59,0.89	0.002	0.000	70%

Abbreviations: CAD, coronary artery disease; CI, confidence interval; LPL, lipoprotein lipase; *N*, number of studies; OR, odds ratio; *P*_-Value_, *P* value for association; *P*_Heterogeneity_, *P* value for heterogeneity.

### Association between the *LPL* S477X polymorphism and susceptibility to CAD

**Figure 3:**
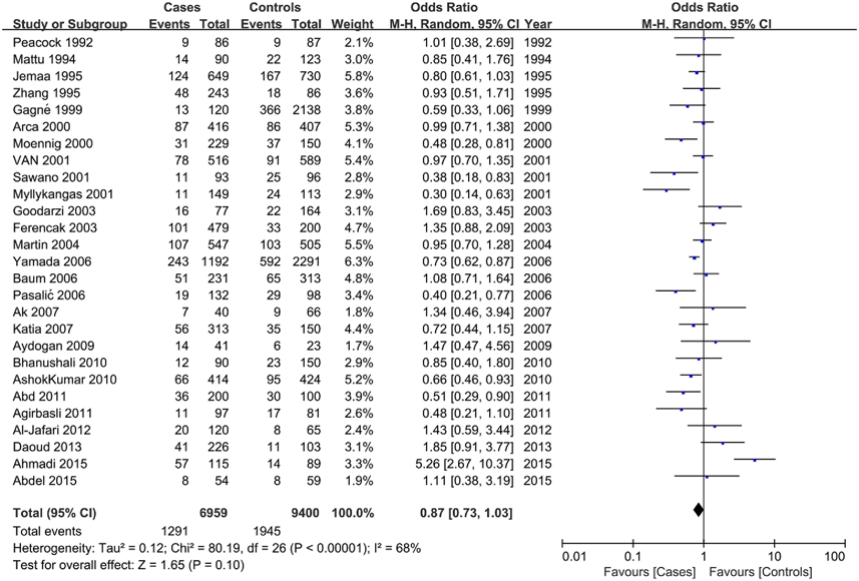
Forest plot of odds ratios for the association between the *LPL* S447X polymorphism and CAD risk under the dominant genetic model (GG + GC vs. CC)

No significant association was observed in any genetic model between the S477X polymorphism and CAD risk in the overall meta-analysis, and there was some evidence of interstudy heterogeneity (Table 2; Figure 3). The subgroup analysis stratified by ethnicity indicated that the S477X polymorphism was significantly associated with CAD risk for Caucasians, but not Asians, under the dominant and allele genetic models (GG + GC vs. CC: OR = 0.77, 95% CI = 0.64–0.93; GG vs. GC + CC: OR = 0.83, 95% CI = 0.72–0.94), with a reduction in interstudy heterogeneity (Table 2; Supplementary Figure S3). Stratification by sample size indicated that large sample size, but not small sample size, showed a reduced risk of CAD susceptibility associated with the S447X polymorphism under the recessive genetic model (GG vs. GC + CC: OR = 0.55, 95% CI = 0.35–0.86) (Table 2; Supplementary Figure S4).

**Table 2:** Summary of odds ratios (95% CI) in the analysis of the association between the *LPL* S447X polymorphism and CAD susceptibility

Genetic model	Overall and subgroups	*N*	Test of association			Test of heterogeneity	
			OR	95% CI	*P*-value	*P*_Heterogeneity_	*I*^2^ (%)
**GG + GC vs. CC**	Overall	27	0.87	0.73,1.03	0.100	0.000	68%
	Asians	8	1.08	0.71,1.65	0.730	0.000	84%
	Caucasians	16	0.77	0.64,0.93	0.008	0.007	52%
	Large sample	9	0.87	0.75,1.00	0.050	0.080	44%
	Small sample	18	0.87	0.62,1.22	0.430	0.000	74%
**GG vs. GC + CC**	Overall	19	1.00	0.60,1.68	1.000	0.040	40%
	Asians	6	0.90	0.30,2.69	0.850	0.002	74%
	Caucasians	11	0.78	0.45,1.35	0.370	0.730	0%
	Large sample	6	0.55	0.35,0.86	0.009	0.950	0%
	Small sample	13	1.59	0.81,3.11	0.180	0.200	24%
**G vs. C**	Overall	21	0.94	0.77,1.15	0.540	0.000	76%
	Asians	8	1.11	0.72,1.72	0.630	0.000	88%
	Caucasians	11	0.83	0.72,0.94	0.005	0.020	53%
	Large sample	6	0.87	0.74,1.02	0.080	0.080	50%
	Small sample	15	0.97	0.67,1.41	0.880	0.000	80%

Abbreviations: CAD, coronary artery disease; CI, confidence interval; LPL, lipoprotein lipase; *N*, number of studies; OR, odds ratio; *P*_-Value_, *P* value for association; *P*_Heterogeneity_, *P* value for heterogeneity.

### Association between the *LPL* N291S and D9N gene polymorphisms and susceptibility to CAD

**Figure 4:**
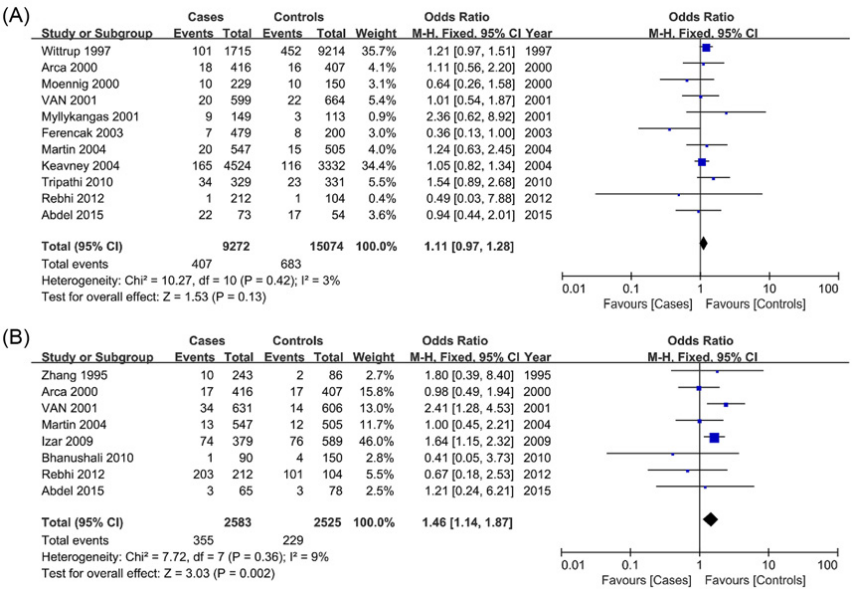
Forest plot of odds ratios for the association of polymorphisms in *LPL* N291S and D9N and susceptibility to CAD (**A**) The N291S polymorphism under the dominant genetic model (GG + GA vs. AA). (**B**) The D9N polymorphism under the dominant genetic model (AA+GA vs. GG).

Because of the low number of minor homozygotes, only the dominant genetic model was applied to the N291S and D9N polymorphisms. An increased risk of CAD susceptibility was associated with the D9N polymorphism under the dominant genetic model (AA + GA vs. GG: OR = 1.46, 95% CI = 1.14–1.87) with low interstudy heterogeneity (Table 3; Figure 4). No significant association was observed between the N291S polymorphism and CAD risk (Table 3; Figure 4). When we conducted subgroup analyses by ethnicity and sample size, the same significant association was observed in large sample size of the D9N polymorphism (Table 3; Supplementary Figure S5). However, no significant association was observed between CAD risk and the N291S polymorphism in the subgroup analysis (Table 3; Supplementary Figure S6).

**Table 3:** Summary of odds ratios (95% CI) in the analysis of the association between the *LPL* N291S and D9N polymorphisms and CAD susceptibility

Genetic model	Overall and subgroups	*N*	Test of association			Test of heterogeneity	
			OR	95% CI	*P*-value	*P*_Heterogeneity_	*I*^2^ (%)
**N291S**							
**GG + GA vs. AA**	Overall	11	1.11	0.97,1.28	0.130	0.420	3%
	Asians	1	1.54	0.89,2.68	0.120	N/A	N/A
	Caucasians	8	1.10	0.95,1.27	0.210	0.300	16%
	Large sample	7	1.13	0.98,1.30	0.100	0.320	15%
	Small sample	4	0.96	0.57,1.60	0.860	0.430	0%
**D9N**							
**AA + GA vs. GG**	Overall	8	1.46	1.14,1.87	0.002	0.360	9%
	Asians	1	0.41	0.05,3.73	0.430	N/A	N/A
	Caucasians	4	1.47	1.00,2.14	0.050	0.200	36%
	Large sample	4	1.49	1.03,2.15	0.040	0.180	38%
	Small sample	4	0.94	0.42,2.10	0.890	0.670	0%

Abbreviations: CAD, coronary artery disease; CI, confidence interval; LPL, lipoprotein lipase; *N*, number of studies; N/A, not applicable; OR, odds ratio; *P*_-Value_, *P* value for association; *P*_Heterogeneity_, *P* value for heterogeneity.

### Association between the *LPL* PvuII polymorphism and susceptibility to CAD

**Figure 5:**
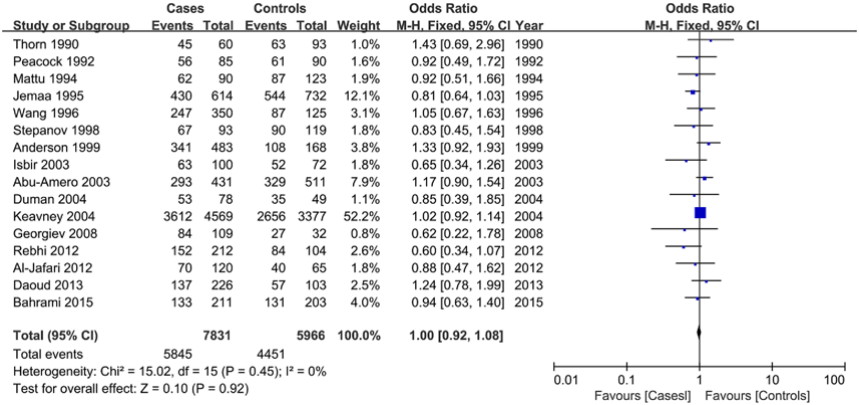
Forest plot of odds ratios for the association between the *LPL* PvuII polymorphism and CAD risk under the dominant model (TT + CT vs. CC)

No significant associations were observed between the *LPL* PvuII polymorphism and CAD susceptibility in any genetic model (Table 4; Figure 5). This was also the case in the subgroup analysis (Table 4; Supplementary Figures S7 and S8).

**Table 4:** Summary of odds ratios (95% CI) in the analysis of the association between the *LPL* PvuII polymorphism and CAD susceptibility

Genetic model	Overall and subgroups	*N*	Test of association			Test of heterogeneity	
			OR	95% CI	*P*-value	*P*_Heterogeneity_	*I^2^* (%)
**TT + CT vs. CC**	Overall	16	1.00	0.92,1.08	0.920	0.450	0%
	Asians	4	1.09	0.90,1.33	0.360	0.660	0%
	Caucasians	11	0.99	0.91,1.08	0.810	0.480	0%
	Large sample	4	1.03	0.87,1.23	0.700	0.080	55%
	Small sample	12	0.92	0.78,1.09	0.320	0.790	0%
**TT vs. CC + CT**	Overall	16	0.90	0.78,1.04	0.150	0.100	32%
	Asians	4	0.95	0.74,1.22	0.670	0.670	0%
	Caucasians	11	0.85	0.69,1.05	0.120	0.030	51%
	Large sample	4	0.98	0.82,1.16	0.780	0.140	45%
	Small sample	12	0.82	0.68,1.00	0.040	0.290	16%
**T vs. C**	Overall	16	0.99	0.94,1.04	0.670	0.200	22%
	Asians	4	1.03	0.90,1.17	0.680	0.490	0%
	Caucasians	11	0.94	0.84,1.04	0.210	0.110	37%
	Large sample	4	1.01	0.89,1.14	0.900	0.040	65%
	Small sample	12	0.90	0.81,1.01	0.060	0.780	0%

Abbreviations: CAD, coronary artery disease; CI, confidence interval; LPL, lipoprotein lipase; *N*, number of studies; OR, odds ratio; *P*_-Value_, *P* value for association; *P*_Heterogeneity_, *P* value for heterogeneity.

### Cumulative analysis

**Figure 6:**
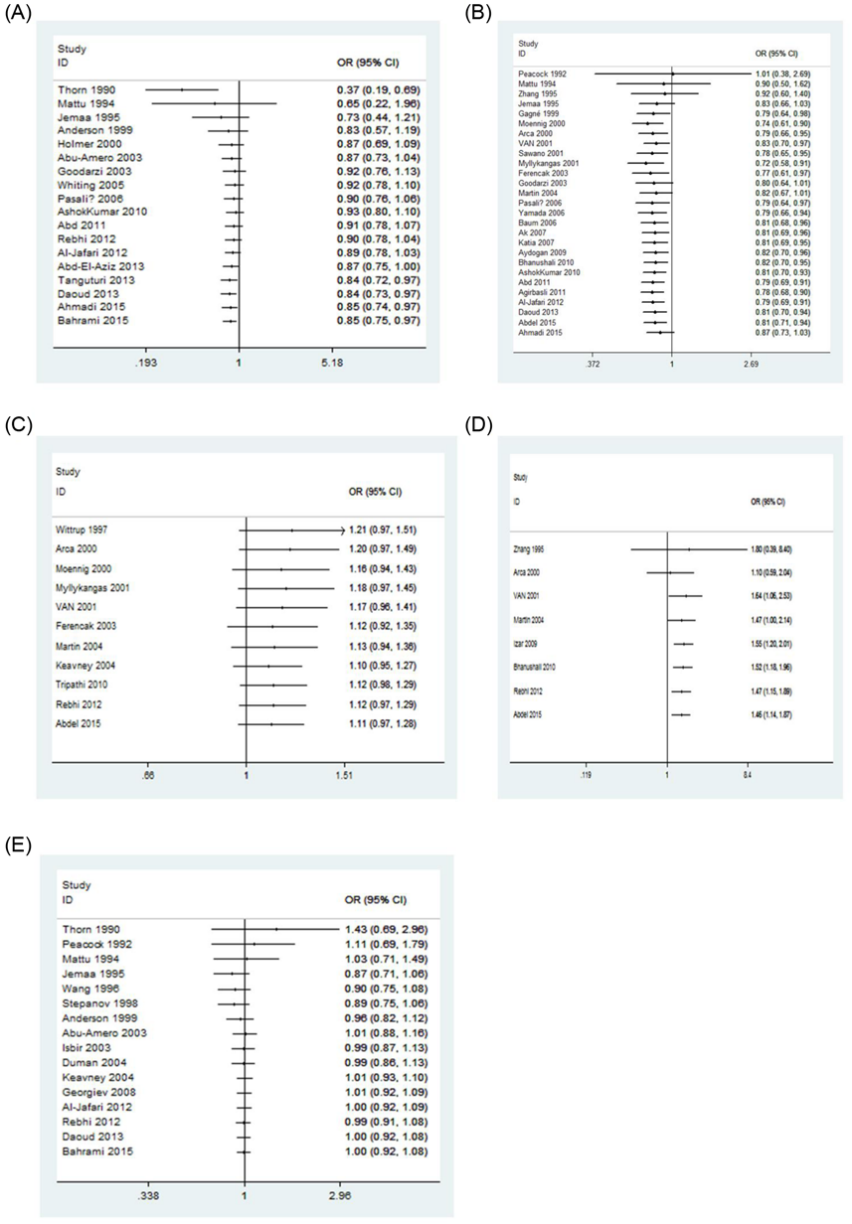
Forest plots of the cumulative odds ratio for the association between the *LPL* gene polymorphisms and CAD risk under the dominant genetic model (**A**) HindIII polymorphism; (**B**) S447X polymorphism; (**C**) N291S polymorphism; (**D**) D9N polymorphism; (**E**) PvuII polymorphism.

For the *LPL* HindIII and D9N polymorphisms, the cumulative meta-analysis showed that as publication year increased, the CI became increasingly narrower, and statistical significance was more common. The association between the *LPL* S447X polymorphism and CAD risk appeared to fluctuate with the number of studies accumulated. For the *LPL* N291S and PvuII polymorphisms, no significant association was observed with the number of studies accumulated (Figure 6).

### Heterogeneity and sensitivity analysis

The heterogeneity within each study in each comparison is shown in Tables 1–4. The influence of each study on the overall meta-analysis was evaluated by deleting one study at a time. The results indicated that no individual study influenced the pooled OR significantly (Supplementary Figure S9).

### Publication bias

**Figure 7:**
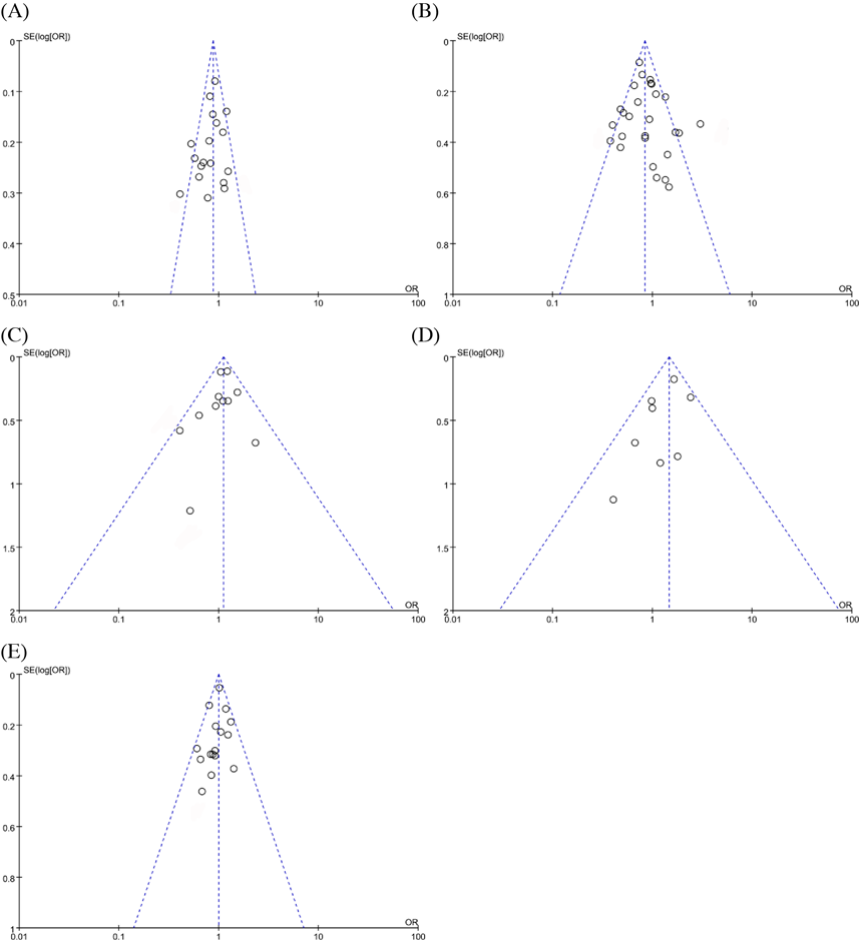
Funnel plot of publication bias for the association between *LPL* gene polymorphisms and susceptibility to CAD under dominant models (**A**) Hind polymorphism; (**B**) S447X polymorphism; (**C**) N291S polymorphism; (**D**) D9N polymorphism; (**E**) PvuII polymorphism.

The Funnel plot and Egger’s regression test were applied to assess the publication bias of the included studies. The results indicated that the distribution of the included studies on the funnel plot appeared roughly symmetrical (Figure 7). The results of Egger’s regression test are also presented under the dominant models (HindIII: *t* = −1.23, *P*=0.237; S477X: *t* = −3.12, *P*=0.005; N291S: *t* = −0.96, *P*=0.363; D9N: *t* = −1.62, *P*=0.157; PvuII: *t* = −1.05, *P*=0.311) (Supplementary Figure S10).

## Discussion

Genetic variations in the *LPL* gene could influence lipid transport and metabolism and could consequently modulate an individual’s susceptibility to atherosclerosis. However, it is difficult to draw a definite conclusion for whether LPL is a proatherosclerotic or antiatherosclerotic factor, since the effects of LPL partly depend on its locations and activity [58]. The enzyme, when expressed in adipose tissue, heart, and skeletal muscle, has been regarded as an antiatherosclerotic factor by reducing atherogenic lipoproteins or increasing HDL, whereas the effect of LPL on the biology of arterial wall seems to be atherogenic by accelerating lipid accumulation [58,59]. Several lines of evidence also suggest that LPL activity is higher in atherosclerotic arteries compared with normal arteries [10,60]. Increased plasma LPL activity could alter lipid traits, such as decreasing TG and increasing HDL levels, generating a profile associated with protection against atherosclerosis, while the down-regulation of *LPL* gene expression has been shown to play an opposite role [61,62].

Although numerous studies have investigated the correlation between LPL and CAD risk in the past several decades, no definite conclusions have been reached regarding gene polymorphisms. This meta-analysis has combined and reanalyzed individual participant data from 80 eligible studies of the effect of *LPL* gene polymorphisms on CAD incidence. In our study, all of the results revealed that three *LPL* gene variants (Hind III, S447X, and D9N) were associated with CAD susceptibility. When Asians or Caucasians were analyzed independently, the heterogeneity of the population tended to be weaker, and the subgroup analysis indicated that S447X polymorphism decreased CAD risk in Caucasians. On the other hand, the stratified analysis by ethnicity for the S447X polymorphism was successfully applied to relieve the heterogeneity bias in the polymorphism analysis within Caucasians, suggesting that ethnicity may potentially be the source of the heterogeneity. In addition, it is worth noting that the *P* value of the Egger’s regression test for the S447X polymorphism was less than 0.05, which indicated that publication bias likely existed; however, the funnel plot appeared roughly symmetrical, and the sensitivity analysis indicated the stability of the results. Consequently, future studies are warranted to validate our conclusion.

Although several relevant meta-analyses have been published, our study had certain specific advantages [63–65]. Compared with other studies, we incorporated more eligible articles, conducted quality assessment, and performed a comprehensive analysis, whereas previous studies primarily focused on the plasma levels of lipids and lipoproteins, or they only analyzed a single gene variant in the meta-analysis. Furthermore, in the present study, a cumulative meta-analysis was performed to assess the pattern of the evidence accumulated over time.

Several limitations in our study should also be addressed. First, some genetic models displayed high heterogeneity, although subgroup analysis was performed to detect the sources of this heterogeneity. Second, the ethnic distribution of included studies was primarily Asians and Caucasians. Racial bias may exist, and the conclusions may not be applicable to other races. Third, we searched and collected articles in English from four comprehensive electronic databases, including PubMed, Web of Science, EMBASE, and Cochrane database. Several publications related to this topic written in other languages might have been ignored. Thus, publication bias likely existed. However, the articles included in these four databases are more authoritative and more convenient for readers compared with the original literature.

In summary, this updated meta-analysis suggested that the *LPL* D9N polymorphism was associated with the increased risk of CAD, whereas the *LPL* HindIII and S447X polymorphisms showed protective effects against CAD. No associations were observed between the *LPL* N291S and PvuII polymorphisms and susceptibility to CAD.

## Supporting information

**Supplementary Table 1.** MOOSE checklist for meta-analysis of observational studies.

**Supplementary Table 2.** Characteristics of the individual studies included in the meta-analysis.

**Supplementary Table 3.** Methodological quality of the selected studies according to the Newcastle-Ottawa Scale.

**supplementary Figure 1:** Stratified analysis based on ethnicity for the association between the *LPL* HindIII polymorphism and CAD risk using dominant genetic model (GG+GT vs. TT).

**supplementary Figure 2:** Stratified analysis based on sample size for the association between the *LPL* HindIII polymorphism and CAD risk using dominant genetic model (GG+GT vs. TT).

**supplementary Figure 3:** Stratified analysis based on ethnicity for the association between the *LPL* S447X polymorphism and CAD risk using dominant genetic model (GG+GC vs. CC).

**supplementary Figure 4:** Stratified analysis based on sample size for the association between the *LPL* S447X polymorphism and CAD risk using dominant genetic model (GG+GC vs. CC).

**supplementary Figure 5:** Stratified analysis based on sample size for the association between the *LPL* D9N polymorphism and CAD risk using dominant genetic model (AA+GA vs. GG).

**supplementary Figure 6:** Stratified analysis based on sample size for the association between the *LPL* N291S polymorphism and CAD risk using dominant genetic model (GG+GA vs. AA).

**supplementary Figure 7:** Stratified analysis based on ethnicity for the association between the *LPL* PvuII polymorphism and CAD risk using dominant genetic model (TT+CT vs. CC).

**supplementary Figure 8:** Stratified analysis based on sample size for the association between the *LPL* PvuII polymorphism and CAD risk using dominant genetic model (TT+CT vs. CC).

**supplementary Figure 9:** Egger’s regression test of publication bias for the association between the *LPL* gene polymorphisms and susceptibility to CAD. (a). HindIII polymorphism; (b). S447X polymorphism; (c). N291S polymorphism; (d). D9N polymorphism; (e). PvuII polymorphism.

**supplementary Figure 10:** Sensitivity analysis on the correlation between the *LPL* gene polymorphisms and susceptibility to CAD. (a). sensitivity analysis for HindIII and CAD risk; (b). sensitivity analysis for S447X and CAD risk; (c). sensitivity analysis for N291S and CAD risk; (d). sensitivity analysis for D9N and CAD risk; (e). sensitivity analysis for PvuII and CAD risk;

## Abbreviations

CAD: coronary artery disease
CM: chylomicrons
CI: confidence interval
GWAS: genome-wide association studies
HDL-C: high-density lipoprotein cholesterol
HWE: Hardy–Weinberg equilibrium
LDL-C: low-density lipoprotein cholesterol
LPL: lipoprotein lipase
NOS: Newcastle–Ottawa scale
OR: odds ratio
TC: cholesterol
TG: triglyceride
TRL: triglyceride-rich lipoprotein
VLDL: very-low-density lipoprotein
