# Supplementary material for: Associations between LPL gene polymorphisms and coronary artery disease: evidence based on an updated and cumulative meta-analysis
Source: Biosci Rep. 2018 Mar 16;38(2):BSR20171642. doi: 10.1042/BSR20171642 (PMC5857905; doi:10.1042/BSR20171642)
Supplement: Supplementary file 1 [file bsr20171642_Supp1.pdf]

Associations between *LPL* gene polymorphisms and coronary artery disease: evidence based on an updated and cumulative meta-analysis

Short title: *LPL* gene polymorphisms and coronary artery disease

Wen-Qi Ma[1], Ying Wang[1], Xi-Qiong Han[1], Yi Zhu[1], Nai-Feng Liu[1*]

[1] Department of Cardiology, Zhongda Hospital, School of Medicine, Southeast University 87 Dingjiaqiao, Nanjing 210009, P.R. China.

* Corresponding author: Nai-Feng Liu; E-mail: liunf@seu.edu.cn

Supplementary Information

Supplementary Table 1. MOOSE checklist for meta-analysis of observational studies.

Supplementary Table 2. Characteristics of the individual studies included in the meta-analysis.

Supplementary Table 3. Methodological quality of the selected studies according to the Newcastle-Ottawa Scale.

Supplementary Figure 1. Stratified analysis based on ethnicity for the association between the *LPL* HindIII polymorphism and CAD risk using dominant genetic model (GG+GT vs. TT).

Supplementary Figure 2. Stratified analysis based on sample size for the association between the *LPL* HindIII polymorphism and CAD risk using dominant genetic model (GG+GT vs. TT).

Supplementary Figure 3. Stratified analysis based on ethnicity for the association between the *LPL* S447X polymorphism and CAD risk using dominant genetic model (GG+GC vs. CC).

Supplementary Figure 4. Stratified analysis based on sample size for the association between the *LPL* S447X polymorphism and CAD risk using dominant genetic model (GG+GC vs. CC).

Supplementary Figure 5. Stratified analysis based on sample size for the association between the *LPL* D9N polymorphism and CAD risk using dominant genetic model (AA+GA vs. GG).

Supplementary Figure 6. Stratified analysis based on sample size for the association between the *LPL* N291S polymorphism and CAD risk using dominant genetic model (GG+GA vs. AA).

Supplementary Figure 7. Stratified analysis based on ethnicity for the association between the *LPL* PvuII polymorphism and CAD risk using dominant genetic model (TT+CT vs. CC).

Supplementary Figure 8. Stratified analysis based on sample size for the association between the *LPL* PvuII polymorphism and CAD risk using dominant genetic model (TT+CT vs. CC).

Supplementary Figure 9. Egger's regression test of publication bias for the association between the *LPL* gene polymorphisms and susceptibility to CAD. (a). HindIII polymorphism; (b). S447X polymorphism; (c). N291S polymorphism; (d). D9N polymorphism; (e). PvuII polymorphism.

Supplementary Figure 10. Sensitivity analysis on the correlation between the *LPL* gene polymorphisms and susceptibility to CAD. (a). sensitivity analysis for HindIII and CAD risk; (b). sensitivity analysis for S447X and CAD risk; (c). sensitivity analysis for N291S and CAD risk; (d). sensitivity analysis for D9N and CAD risk; (e). sensitivity analysis for PvuII and CAD risk;

Supplementary Table 1. MOOSE checklist for meta-analysis of observational studies.

Item No	Recommendation	Reported on Page No
Reporting of background should include		
1	Problem definition	2-3
2	Hypothesis statement	2-3
3	Description of study outcome(s)	2-3
4	Type of exposure or intervention used	2-3
5	Type of study designs used	2-3
6	Study population	2-3
Reporting of search strategy should include		
7	Qualifications of searchers (eg, librarians and investigators)	4
8	Search strategy, including time period included in the synthesis and key words	4
9	Effort to include all available studies, including contact with authors	4
10	Databases and registries searched	4
11	Search software used, name and version, including special features used (eg, explosion)	5
12	Use of hand searching (eg, reference lists of obtained articles)	4
13	List of citations located and those excluded, including justification	4-5
14	Method of addressing articles published in languages other than English	4
15	Method of handling abstracts and unpublished studies	5
16	Description of any contact with authors	No
Reporting of methods should include		
17	Description of relevance or appropriateness of studies assembled for assessing the hypothesis to be tested	4-5
18	Rationale for the selection and coding of data (eg, sound clinical principles or convenience)	4-5
19	Documentation of how data were classified and coded (eg, multiple raters, blinding and interrater reliability)	4-6
20	Assessment of confounding (eg, comparability of cases and controls in studies where appropriate)	4-6
21	Assessment of study quality, including blinding of quality assessors, stratification or regression on possible predictors of study results	4-6
22	Assessment of heterogeneity	5-6
23	Description of statistical methods (eg, complete description of fixed or random effects models, justification of whether the chosen models account for predictors of study results, dose-response models, or cumulative meta-analysis) in sufficient detail to be replicated	5-6
24	Provision of appropriate tables and graphics	5-6
Reporting of results should include		
25	Graphic summarizing individual study estimates and overall estimate	6-9
26	Table giving descriptive information for each study included	Table1
27	Results of sensitivity testing (eg, subgroup analysis)	6-14
28	Indication of statistical uncertainty of findings	6-9
Reporting of discussion should include		
29	Quantitative assessment of bias (eg, publication bias)	10-11
30	Justification for exclusion (eg, exclusion of non-English language citations)	10-11
31	Assessment of quality of included studies	14-16
Reporting of conclusions should include		
32	Consideration of alternative explanations for observed results	10-11
33	Generalization of the conclusions (ie, appropriate for the data presented and within the domain of the literature review)	10-11
34	Guidelines for future research	11
35	Disclosure of funding source	11

Supplementary Table 2. Characteristics of the individual studies included in the meta-analysis.

First Author /Year	Country	Disease	Source of Controls	Gene-typing Methods	Sample Size	Genotype Distribution		P_{HWE}	NOS
					Cases/Controls	Cases	Controls		
**Hind III**						**TT/TG/GG**	**TT/TG/GG**		
Thorn 1990	UK	CAD	PB	PCR	63/108	37/23/3	37/51/20	0.743	7
Mattu 1994	Welsh	CAD	PB	PCR-RFLP	90/123	50/34/6	72/45/6	0.760	8
Jemaa 1995	France	CAD	PB	PCR	614/733	318/258/38	343/314/76	0.742	7
Anderson 1999	America	CAD	HB	PCR	483/168	259/194/30	94/52/22	>0.05	7
Holmer 2000	Germany	MI	PB	PCR	1159/1361	616/456/87	697/564/100	0.332	8
Abu-Amero 2003	Saudi Arabia	CAD	HB	PCR	352/410	189/138/25	206/173/31	0.518	6
Goodarzi 2003	USA	CAD	PB	PCR	77/164	39/33/5	105/52/7	0.861	7
Whiting 2005	America	CAD	HB	PCR	713/196	385/269/59	103/77/16	0.763	7
Pasalić 2006	Croatia	CAD	HB	PCR-RFLP	132/98	78/46/8	47/45/6	0.262	7
AshokKumar 2010	India	CAD	HB	PCR	414/424	220/168/26	245/158/21	0.486	7
Abd 2011	Egypt	MI	HB	PCR	200/100	120/70/10	50/36/14	0.834	7
Al-Jafari 2012	Saudi Arabia	CAD	HB	PCR	120/65	61/53/6	29/23/13	0.050	7
Rebhi 2012	Tunisia	CAD	HB	PCR–RFLP	212/104	114/83/15	47/39/18	0.569	7
Abd-El-Aziz 2013	Egypt	CAD	HB	PCR-RFLP	156/154	100/53/3	78/54/22	>0.05	7
Tanguturi 2013	India	MI	PB	PCR	202/210	98/72/32	70/68/72	>0.05	7
Daoud 2013	Saudi Arabia	CAD	HB	PCR-RFLP	226/103	102/81/43	42/35/26	>0.05	7
Ahmadi 2015	Iran	CAD	HB	PCR-RFLP	108/89	61/41/6	53/33/3	0.430	6
Bahrami 2015	Iran	MI	HB	PCR-RFLP	211/203	116/81/14	101/83/19	0.745	7
**S477X**						**CC/CG/GG**	**CC/CG/GG**		
Peacock 1992	Sweden	CAD	HB	PCR	86/87	77/9 [a]	78/9 [a]	>0.05	7
Mattu 1994	Welsh	CAD	PB	PCR-RFLP	90/123	76/14/0	101/21/1	0.936	8
Jemaa 1995	France	CAD	PB	PCR	649/730	525/118/6	563/154/13	0.514	7
Zhang 1995	Germany	CAD	HB	PCR	243/86	195/46/2	68/17/1	0.959	7
Gagné 1999	America	CAD	PB	PCR-RFLP	120/2138	107/13 [a]	1772/366 [a]	>0.05	7
Arca 2000	Italy	CAD	HB	PCR-RFLP	416/407	329/87 [a]	321/86 [a]	>0.05	6
Moennig 2000	Germany	CAD	PB	PCR	229/150	198/28/3	113/37/0	0.085	8
Sawano 2001	Japan	CAD	PB	PCR	93/96	82/10/1	71/23/2	0.932	8
VAN 2001	Australia.	CAD	PB	PCR	516/589	438/78 [a]	498/91 [a]	>0.05	7
Myllykangas 2001	Finland	CAD	HB	PCR	149/113	138/11 [a]	89/24 [a]	>0.05	7
Ferencak 2003	Croatia	CAD	HB	PCR	479/200	378/97/4	167/32/1	0.686	7

Goodarzi 2003	USA	CAD	PB	PCR	77/164	61/15/1	142/22/0	>0.05	7
Martin 2004	UK	MI	HB	PCR-RFLP	547/505	440/104/3	402/99/4	0.483	7
Baum 2006	China	MI	HB	PCR	231/313	180/51/0	248/64/1	0.137	6
Pasalić 2006	Croatia	CAD	HB	PCR-RFLP	132/98	113/19/0	69/28/1	0.312	7
Yamada 2006	Japan	MI	HB	PCR	1192/2291	949/231/12	1699/547/45	0.900	7
Ak 2007	Turkey	CAD	HB	PCR	40/66	33/7/0	57/8/1	0.275	6
Katia 2007	Brazil	CAD	PB	PCR-RFLP	313/150	257/47/9	115/34/1	0.110	7
Aydogan 2009	Turkey	CAD	PB	PCR-RFLP	41/23	27/4/10	17/4/2	0.058	8
AshokKumar 2010	India	CAD	HB	PCR	414/424	348/62/4	329/87/8	0.427	7
Bhanushali 2010	India	CAD	HB	PCR	90/150	78/11/1	127/21/2	0.306	7
Abd 2011	Egypt	MI	HB	PCR	200/100	164/32/4	70/26/4	0.431	7
Agirbasli 2011	Turkey	CAD	HB	PCR-RFLP	97/81	86/10/1	64/16/1	1.000	7
Al-Jafari 2012	Saudi Arabia	CAD	HB	PCR	120/65	100/20/0	57/8/0	0.597	7
Daoud 2013	Saudi Arabia	CAD	HB	PCR-RFLP	226/103	185/41/0	92/11/0	0.567	7
Ahmadi 2015	Iran	CAD	HB	PCR-RFLP	115/89	58/23/34	75/7/7	>0.05	6
Abdel 2015	Sudan	CAD	HB	PCR-RFLP	54/59	46/8 [a]	51/8 [a]	>0.05	5
**N291S**						**AA/AG/GG**	**AA/AG/GG**		
Wittrup 1997	Danish	IHD	PB	PCR	1715/9214	1614/101 [b]	8762/452 [b]	>0.05	8
Arca 2000	Italy	CAD	HB	PCR-RFLP	416/407	398/18/0	391/16/0	0.686	6
Moennig 2000	Germany	CAD	PB	PCR	229/150	219/10/0	140/10/0	0.673	8
VAN 2001	Australia.	CAD	PB	PCR	599/664	579/20 [b]	642/22 [b]	>0.05	7
Myllykangas 2001	Finland	CAD	HB	PCR	149/113	140/9 [b]	110/3 [b]	>0.05	7
Ferencak 2003	Croatia	CAD	HB	PCR	479/200	472/7/0	192/8/0	0.773	7
Martin 2004	UK	MI	HB	PCR-RFLP	547/505	527/20/0	490/15/0	0.242	7
Keavney 2004	UK	MI	PB	PCR	4524/3332	4359/162/3	3216/112/4	>0.05	7
Tripathi 2010	India	CAD	HB	PCR	329/331	295/34/0	308/23/0	0.513	6
Rebhi 2012	Tunisia	CAD	HB	PCR–RFLP	212/104	211/1/0	103/1/0	>0.05	7
Abdel 2015	Sudan	CAD	HB	PCR-RFLP	73/54	51/22 [b]	37/17 [b]	>0.05	5
**D9N**						**GG/GA/AA**	**GG/GA/AA**		
Zhang 1995	Germany	CAD	HB	PCR	243/86	233/10/0	84/2/0	0.913	7
Arca 2000	Italy	CAD	HB	PCR-RFLP	416/407	382/17/0	373/17/0	0.660	6
VAN 2001	Australia.	CAD	PB	PCR	631/606	597/34 [c]	592/14 [c]	>0.05	7
Martin 2004	UK	MI	HB	PCR-RFLP	547/505	534/13/0	493/12/0	0.787	7
Izar 2009	Brazil	MI	PB	PCR-RFLP	379/583	305/71/3	507/73/3	0.832	8
Bhanushali 2010	India	CAD	HB	PCR	90/150	89/1/0	146/4/0	0.869	7
Rebhi 2012	Tunisia	CAD	HB	PCR–RFLP	212/104	9/47/156	3/17/84	0.848	7
Abdel 2015	Sudan	CAD	HB	PCR-RFLP	65/78	62/3 [c]	75/3 [c]	>0.05	5
**Pvull**						**CC/CT/TT**	**CC/CT/TT**		
Thorn 1990	UK	CAD	PB	PCR	60/93	15/39/6	30/43/20	0.534	7

Peacock 1992	Sweden	CAD	HB	PCR	85/90	29/38/18	29/42/19	0.602	7
Mattu 1994	Welsh	CAD	PB	PCR-RFLP	90/123	28/42/20	36/64/23	0.561	8
Jemaa 1995	France	CAD	PB	PCR	614/732	184/302/128	188/357/187	0.506	7
Wang 1996	Australia	CAD	HB	PCR-RFLP	350/125	103/180/67	38/59/28	0.577	7
Stepanov 1998	Russia	CAD	PB	PCR-RFLP	93/119	26/52/15	29/57/33	0.655	7
Anderson 1999	America	CAD	HB	PCR	483/168	142/236/105	60/76/32	0.368	7
Abu-Amero 2003	Saudi Arabia	CAD	HB	PCR	431/511	138/225/68	182/248/81	0.819	6
Isbir 2003	Turkey	CAD	PB	PCR	100/72	37/49/14	20/40/12	0.289	7
Duman 2004	Turkey	CAD	HB	PCR	78/49	25/39/14	14/16/19	0.017	6
Keavney 2004	UK	MI	PB	PCR	4569/3377	957/2266/1346	721/1694/962	0.626	7
Georgiev 2008	Macedon	CAD	HB	PCR-RFLP	109/32	25/58/26	5/20/7	0.149	7
Al-Jafari 2012	Saudi Arabia	CAD	HB	PCR	120/65	50/52/18	25/28/12	0.408	7
Rebhi 2012	Tunisia	CAD	HB	PCR–RFLP	212/104	60/90/62	20/55/29	0.503	7
Daoud 2013	Saudi Arabia	CAD	HB	PCR-RFLP	226/103	89/102/35	46/44/13	0.627	7
Bahrami 2015	Iran	MI	HB	PCR-RFLP	211/203	78/101/32	72/93/38	0.414	7

Abbreviations: CAD, coronary artery disease; MI, myocardial infarction; PCR, polymerase chain reaction; PCR-RFLP, polymerase chain reaction-restriction fragment length polymorphism; HWE, Hardy–Weinberg equilibrium for controls; NOS, Newcastle–Ottawa quality scale; IHD, ischemic heart disease; PB, population-based control; HB, hospital-based control. Note: a, CC vs. GC+GG; b, AA vs. AG+GG; c, GG vs. AG+AA.

Supplementary Table 3. Methodological quality of the selected studies according to the Newcastle-Ottawa Scale.

Study	Adequacy of Case Definition	Representativeness of the cases	Selection of controls	Definition of controls	Comparability of cases/controls	Ascertainment of exposure	Same method of ascertainment	Non-Response rate
Thorn 1990	★	★	★	★	★	★	★	N/A
Peacock 1992	★	★	N/A	★	★★	★	★	N/A
Mattu 1994	★	★	★	★	★★	★	★	N/A
Jemaa 1995	★	★	★	★	★	★	★	N/A
Zhang 1995	★	★	N/A	★	★★	★	★	N/A
Wang 1996	★	★	N/A	★	★★	★	★	N/A
Wittrup 1997	★	★	★	★	★★	★	★	N/A
Stepanov 1998	★	★	★	★	★	★	★	N/A
Anderson 1999	★	★	N/A	★	★★	★	★	N/A
Gagné 1999	★	★	★	★	★	★	★	N/A
Holmer 2000	★	★	★	★	★★	★	★	N/A
Arca 2000	★	★	N/A	★	★	★	★	N/A
Moennig 2000	★	★	★	★	★★	★	★	N/A
VAN 2001	★	★	★	★	★	★	★	N/A
Myllykangas 2001	★	★	N/A	★	★★	★	★	N/A
Sawano 2001	★	★	★	★	★★	★	★	N/A
Abu-Amero 2003	★	★	N/A	★	★	★	★	N/A
Goodarzi 2003	★	★	★	★	★	★	★	N/A
Ferencak 2003	★	★	N/A	★	★★	★	★	N/A
Isbir 2003	★	★	★	★	★	★	★	N/A
Martin 2004	★	★	N/A	★	★★	★	★	N/A
Keavney 2004	★	★	★	★	★	★	★	N/A
Duman 2004	★	★	N/A	★	★	★	★	N/A
Whiting 2005	★	★	N/A	★	★★	★	★	N/A
Baum 2006	★	★	N/A	★	★	★	★	N/A
Pasalić 2006	★	★	N/A	★	★★	★	★	N/A
Yamada 2006	★	★	N/A	★	★★	★	★	N/A
Ak 2007	★	★	N/A	★	★	★	★	N/A
Katia 2007	★	★	★	★	★	★	★	N/A
Georgiev 2008	★	★	N/A	★	★★	★	★	N/A
Izar 2009	★	★	★	★	★★	★	★	N/A
Aydogan 2009	★	★	★	★	★★	★	★	N/A
AshokKumar 2010	★	★	N/A	★	★★	★	★	N/A
Bhanushali 2010	★	★	N/A	★	★★	★	★	N/A
Tripathi 2010	★	★	N/A	★	★	★	★	N/A
Abd 2011	★	★	N/A	★	★★	★	★	N/A

Agirbasli 2011	★	★	N/A	★	★★	★	★	N/A
Al-Jafari 2012	★	★	N/A	★	★★	★	★	N/A
Rebhi 2012	★	★	N/A	★	★★	★	★	N/A
Abd-El-Aziz 2013	★	★	N/A	★	★★	★	★	N/A
Tanguturi 2013	★	★	★	★	★	★	★	N/A
Daoud 2013	★	★	N/A	★	★★	★	★	N/A
Ahmadi 2015	★	★	N/A	★	★	★	★	N/A
Abdel 2015	★	★	N/A	★	★	N/A	★	N/A
Bahrami 2015	★	★	N/A	★	★★	★	★	N/A

This table identifies ‘high’ quality choices with a ‘star’. A study can be awarded a maximum of 1 star for each numbered item within the Selection and Exposure categories. A maximum of 2 stars can be given for Comparability. ★, Yes; N/A, not applicable.

Study or Subgroup	Cases Events	Cases Total	Controls Events	Controls Total	Weight	Odds Ratio M-H, Random, 95% CI	Year
**Caucasians**							
Thorn 1990	26	63	71	108	3.1%	0.37 [0.19, 0.69]	1990
Mattu 1994	40	90	51	123	3.9%	1.13 [0.65, 1.96]	1994
Jemaa 1995	296	614	390	733	10.4%	0.82 [0.66, 1.01]	1995
Anderson 1999	224	483	74	168	6.9%	1.10 [0.77, 1.56]	1999
Holmer 2000	543	1159	664	1361	12.1%	0.93 [0.79, 1.08]	2000
Whiting 2005	328	713	93	196	7.7%	0.94 [0.69, 1.30]	2005
Pasalić 2006	54	132	51	98	4.2%	0.64 [0.38, 1.08]	2006
Abd 2011	80	200	50	100	4.7%	0.67 [0.41, 1.08]	2011
Abd-El-Aziz 2013	56	156	76	154	5.1%	0.57 [0.36, 0.91]	2013
**Subtotal (95% CI)**		**3610**		**3041**	**58.3%**	**0.81 [0.69, 0.96]**	
Total events	1647		1520				
Heterogeneity: Tau² = 0.03; Chi² = 16.31, df = 8 (P = 0.04); I² = 51%							
Test for overall effect: Z = 2.44 (P = 0.01)							
**Asians**							
Abu-Amero 2003	163	352	204	410	8.5%	0.87 [0.65, 1.16]	2003
AshokKumar 2010	194	414	179	424	8.8%	1.21 [0.92, 1.59]	2010
Al-Jafari 2012	59	120	36	65	3.4%	0.78 [0.43, 1.43]	2012
Daoud 2013	124	226	61	103	4.9%	0.84 [0.52, 1.34]	2013
Tanguturi 2013	104	202	140	210	6.1%	0.53 [0.36, 0.79]	2013
Ahmadi 2015	47	108	36	89	3.7%	1.13 [0.64, 2.00]	2015
Bahrami 2015	95	211	102	203	6.3%	0.81 [0.55, 1.19]	2015
**Subtotal (95% CI)**		**1633**		**1504**	**41.7%**	**0.86 [0.70, 1.07]**	
Total events	786		758				
Heterogeneity: Tau² = 0.04; Chi² = 12.47, df = 6 (P = 0.05); I² = 52%							
Test for overall effect: Z = 1.32 (P = 0.19)							
**Total (95% CI)**		**5243**		**4545**	**100.0%**	**0.84 [0.74, 0.95]**	
Total events	2433		2278				
Heterogeneity: Tau² = 0.03; Chi² = 28.98, df = 15 (P = 0.02); I² = 48%							
Test for overall effect: Z = 2.78 (P = 0.005)							
Test for subgroup differences: Chi² = 0.20, df = 1 (P = 0.66), I² = 0%							

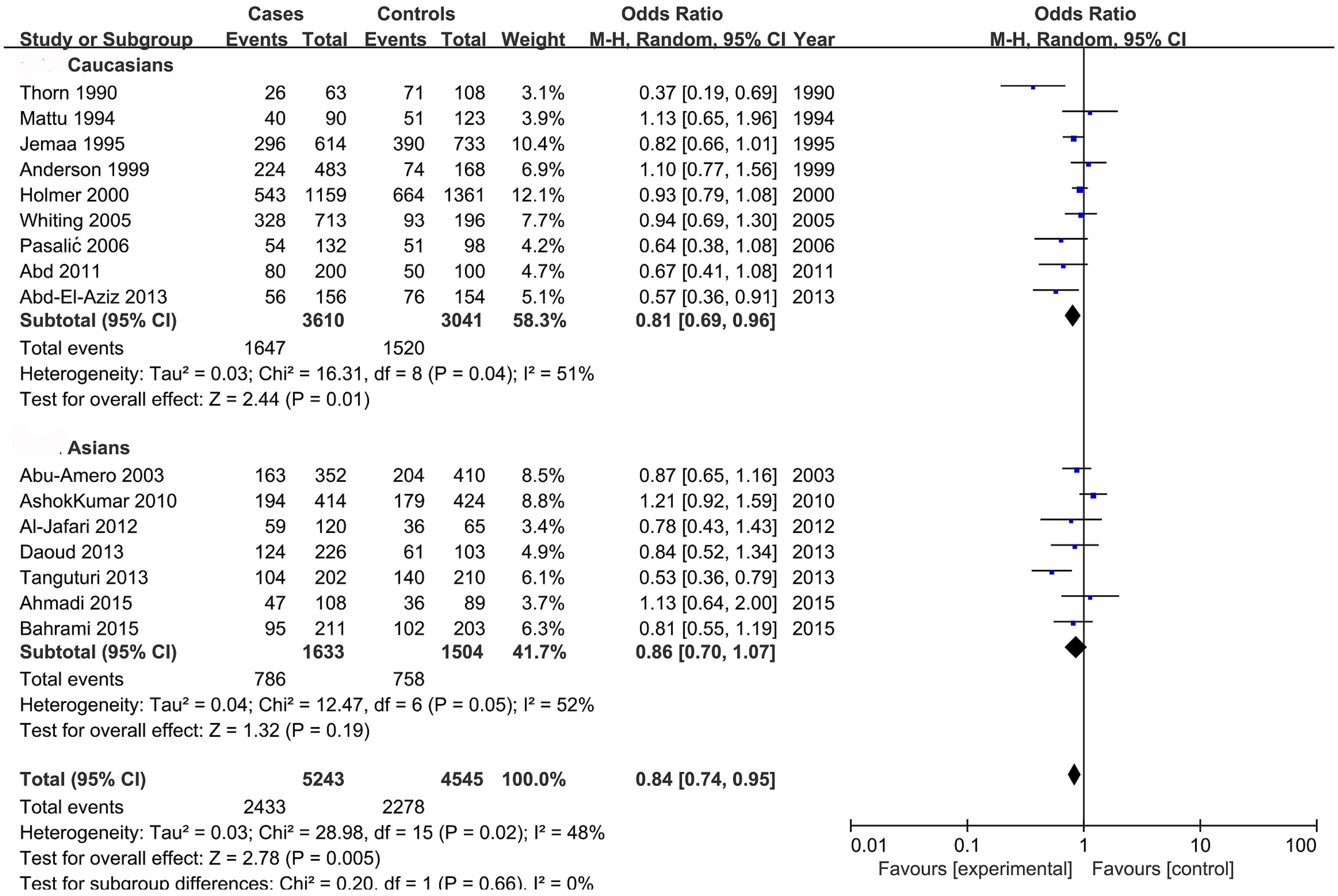

Supplementary Figure 1. Stratified analysis based on ethnicity for the association between the *LPL* HindIII polymorphism and CAD risk using dominant genetic model (GG+GT vs. TT).

	Cases		Controls			Odds Ratio	
Study or Subgroup	Events	Total	Events	Total	Weight	M-H, Random, 95% CI	Year
**large simple sizes**							
Jemaa 1995	296	614	390	733	9.1%	0.82 [0.66, 1.01]	1995
Anderson 1999	224	483	74	168	6.4%	1.10 [0.77, 1.56]	1999
Holmer 2000	543	1159	664	1361	10.3%	0.93 [0.79, 1.08]	2000
Abu-Amero 2003	163	352	204	410	7.6%	0.87 [0.65, 1.16]	2003
Whiting 2005	328	713	93	196	7.0%	0.94 [0.69, 1.30]	2005
AshokKumar 2010	194	414	179	424	7.9%	1.21 [0.92, 1.59]	2010
**Subtotal (95% CI)**		**3735**		**3292**	**48.4%**	**0.94 [0.85, 1.05]**	
Total events	1748		1604				
Heterogeneity: Tau² = 0.00; Chi² = 5.88, df = 5 (P = 0.32); I² = 15%							
Test for overall effect: Z = 1.03 (P = 0.30)							
**small simple sizes**							
Thorn 1990	26	63	71	108	3.0%	0.37 [0.19, 0.69]	1990
Mattu 1994	40	90	51	123	3.8%	1.13 [0.65, 1.96]	1994
Goodarzi 2003	38	77	59	164	3.8%	1.73 [1.00, 3.00]	2003
Pasalić 2006	54	132	51	98	4.0%	0.64 [0.38, 1.08]	2006
Abd 2011	80	200	50	100	4.5%	0.67 [0.41, 1.08]	2011
Rebhi 2012	98	212	57	104	4.6%	0.71 [0.44, 1.14]	2012
Al-Jafari 2012	59	120	36	65	3.3%	0.78 [0.43, 1.43]	2012
Abd-El-Aziz 2013	56	156	76	154	4.8%	0.57 [0.36, 0.91]	2013
Daoud 2013	124	226	61	103	4.6%	0.84 [0.52, 1.34]	2013
Tanguturi 2013	104	202	140	210	5.6%	0.53 [0.36, 0.79]	2013
Ahmadi 2015	47	108	36	89	3.6%	1.13 [0.64, 2.00]	2015
Bahrami 2015	95	211	102	203	5.8%	0.81 [0.55, 1.19]	2015
**Subtotal (95% CI)**		**1797**		**1521**	**51.6%**	**0.76 [0.62, 0.94]**	
Total events	821		790				
Heterogeneity: Tau² = 0.07; Chi² = 23.19, df = 11 (P = 0.02); I² = 53%							
Test for overall effect: Z = 2.58 (P = 0.010)							
**Total (95% CI)**		**5532**		**4813**	**100.0%**	**0.85 [0.75, 0.97]**	
Total events	2569		2394				
Heterogeneity: Tau² = 0.04; Chi² = 35.76, df = 17 (P = 0.005); I² = 52%							
Test for overall effect: Z = 2.49 (P = 0.01)							
Test for subgroup differences: Chi² = 3.30, df = 1 (P = 0.07), I² = 69.7%							

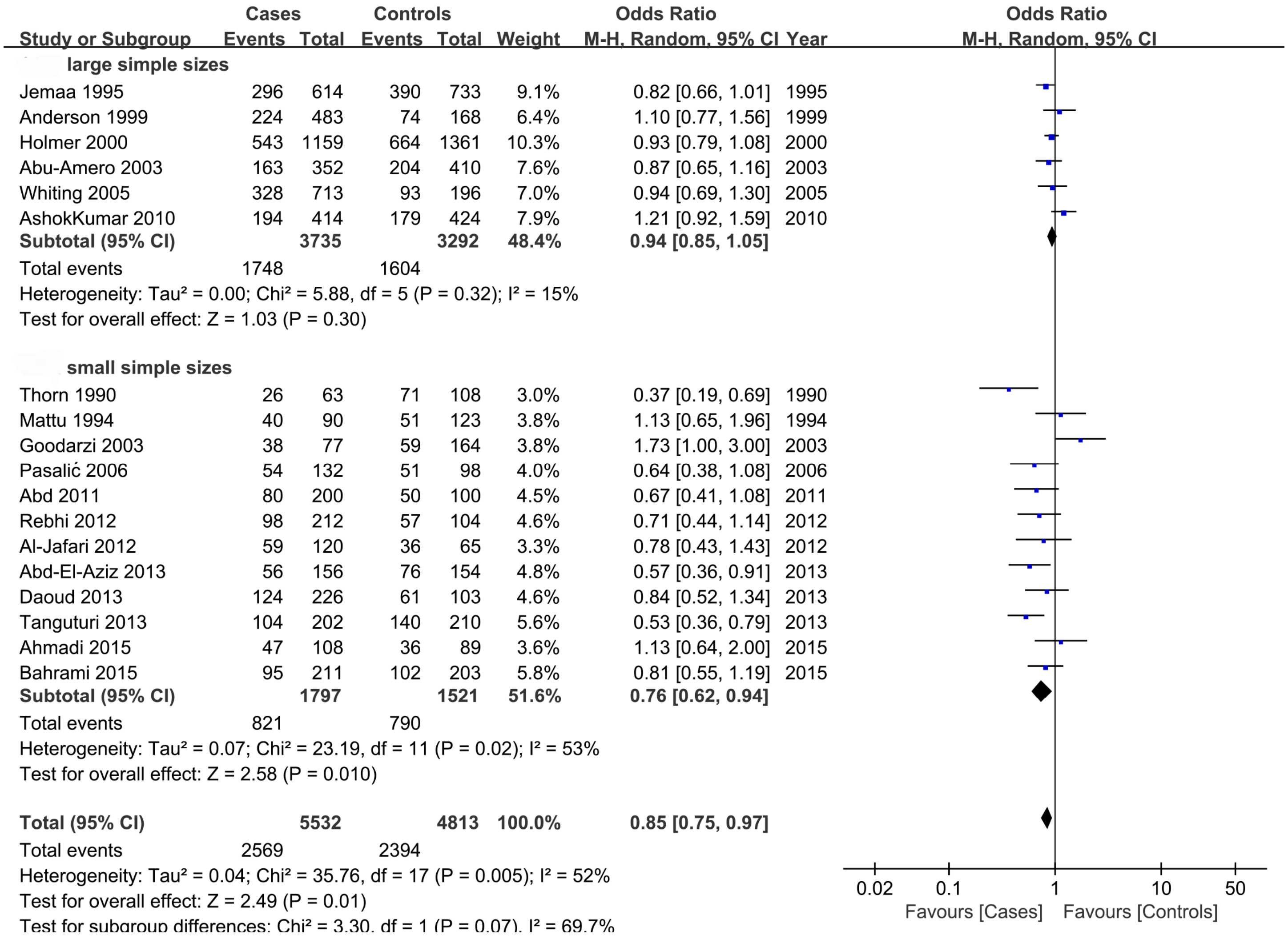

Supplementary Figure 2. Stratified analysis based on sample size for the association between the *LPL* HindIII polymorphism and CAD risk using dominant genetic model (GG+GT vs. TT).

Study or Subgroup	Cases Events	Cases Total	Controls Events	Controls Total	Weight	Odds Ratio M-H, Random, 95% CI	Year
**Caucasians**							
Peacock 1992	9	86	9	87	2.4%	1.01 [0.38, 2.69]	1992
Mattu 1994	14	90	22	123	3.4%	0.85 [0.41, 1.76]	1994
Jemaa 1995	124	649	167	730	6.3%	0.80 [0.61, 1.03]	1995
Zhang 1995	48	243	18	86	4.0%	0.93 [0.51, 1.71]	1995
Gagné 1999	13	120	366	2138	4.2%	0.59 [0.33, 1.06]	1999
Moennig 2000	31	229	37	150	4.5%	0.48 [0.28, 0.81]	2000
Arca 2000	87	416	86	407	5.8%	0.99 [0.71, 1.38]	2000
VAN 2001	78	516	91	589	5.9%	0.97 [0.70, 1.35]	2001
Myllykangas 2001	11	149	24	113	3.2%	0.30 [0.14, 0.63]	2001
Ferencak 2003	101	479	33	200	5.2%	1.35 [0.88, 2.09]	2003
Martin 2004	107	547	103	505	6.1%	0.95 [0.70, 1.28]	2004
Pasalić 2006	19	132	29	98	3.8%	0.40 [0.21, 0.77]	2006
Ak 2007	7	40	9	66	2.1%	1.34 [0.46, 3.94]	2007
Aydogan 2009	14	41	6	23	1.9%	1.47 [0.47, 4.56]	2009
Abd 2011	36	200	30	100	4.3%	0.51 [0.29, 0.90]	2011
Agirbasli 2011	11	97	17	81	2.9%	0.48 [0.21, 1.10]	2011
**Subtotal (95% CI)**		**4034**		**5496**	**65.9%**	**0.77 [0.64, 0.93]**	
Total events	710		1047				
Heterogeneity: Tau² = 0.07; Chi² = 31.56, df = 15 (P = 0.007); I² = 52%							
Test for overall effect: Z = 2.65 (P = 0.008)							
**Asians**							
Sawano 2001	11	93	25	96	3.1%	0.38 [0.18, 0.83]	2001
Yamada 2006	243	1192	592	2291	6.8%	0.73 [0.62, 0.87]	2006
Baum 2006	51	231	65	313	5.3%	1.08 [0.71, 1.64]	2006
Bhanushali 2010	12	90	23	150	3.3%	0.85 [0.40, 1.80]	2010
AshokKumar 2010	66	414	95	424	5.8%	0.66 [0.46, 0.93]	2010
Al-Jafari 2012	20	120	8	65	2.7%	1.43 [0.59, 3.44]	2012
Daoud 2013	41	226	11	103	3.5%	1.85 [0.91, 3.77]	2013
Ahmadi 2015	57	115	14	89	3.6%	5.26 [2.67, 10.37]	2015
**Subtotal (95% CI)**		**2481**		**3531**	**34.1%**	**1.08 [0.71, 1.65]**	
Total events	501		833				
Heterogeneity: Tau² = 0.28; Chi² = 44.10, df = 7 (P < 0.00001); I² = 84%							
Test for overall effect: Z = 0.35 (P = 0.73)							
**Total (95% CI)**		**6515**		**9027**	**100.0%**	**0.85 [0.71, 1.02]**	
Total events	1211		1880				
Heterogeneity: Tau² = 0.12; Chi² = 75.67, df = 23 (P < 0.00001); I² = 70%							
Test for overall effect: Z = 1.76 (P = 0.08)							
Test for subgroup differences: Chi² = 1.99, df = 1 (P = 0.16), I² = 49.9%							

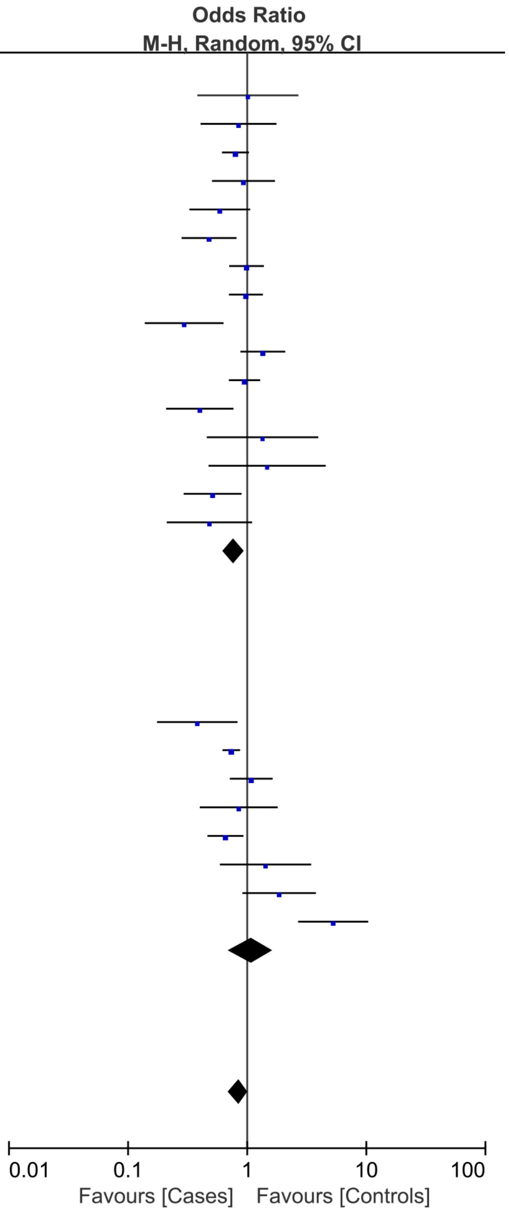

Supplementary Figure 3. Stratified analysis based on ethnicity for the association between the *LPL* S447X polymorphism and CAD risk using dominant genetic model (GG+GC vs. CC).

Study or Subgroup	Cases Events	Cases Total	Controls Events	Controls Total	Weight	Odds Ratio M-H, Random, 95% CI	Year
**large simple sizes**							
Jemaa 1995	124	649	167	730	5.8%	0.80 [0.61, 1.03]	1995
Gagné 1999	13	120	366	2138	3.8%	0.59 [0.33, 1.06]	1999
Arca 2000	87	416	86	407	5.3%	0.99 [0.71, 1.38]	2000
VAN 2001	78	516	91	589	5.4%	0.97 [0.70, 1.35]	2001
Ferencak 2003	101	479	33	200	4.7%	1.35 [0.88, 2.09]	2003
Martin 2004	107	547	103	505	5.5%	0.95 [0.70, 1.28]	2004
Yamada 2006	243	1192	592	2291	6.3%	0.73 [0.62, 0.87]	2006
Baum 2006	51	231	65	313	4.8%	1.08 [0.71, 1.64]	2006
AshokKumar 2010	66	414	95	424	5.2%	0.66 [0.46, 0.93]	2010
**Subtotal (95% CI)**		**4564**		**7597**	**46.8%**	**0.87 [0.75, 1.00]**	
Total events	870		1598				
Heterogeneity: Tau² = 0.02; Chi² = 14.26, df = 8 (P = 0.08); I² = 44%							
Test for overall effect: Z = 1.97 (P = 0.05)							
**small simple sizes**							
Peacock 1992	9	86	9	87	2.1%	1.01 [0.38, 2.69]	1992
Mattu 1994	14	90	22	123	3.0%	0.85 [0.41, 1.76]	1994
Zhang 1995	48	243	18	86	3.6%	0.93 [0.51, 1.71]	1995
Moennig 2000	31	229	37	150	4.1%	0.48 [0.28, 0.81]	2000
Sawano 2001	11	93	25	96	2.8%	0.38 [0.18, 0.83]	2001
Myllykangas 2001	11	149	24	113	2.9%	0.30 [0.14, 0.63]	2001
Goodarzi 2003	16	77	22	164	3.1%	1.69 [0.83, 3.45]	2003
Pasalić 2006	19	132	29	98	3.4%	0.40 [0.21, 0.77]	2006
Katia 2007	56	313	35	150	4.4%	0.72 [0.44, 1.15]	2007
Ak 2007	7	40	9	66	1.8%	1.34 [0.46, 3.94]	2007
Aydogan 2009	14	41	6	23	1.7%	1.47 [0.47, 4.56]	2009
Bhanushali 2010	12	90	23	150	2.9%	0.85 [0.40, 1.80]	2010
Abd 2011	36	200	30	100	3.9%	0.51 [0.29, 0.90]	2011
Agirbasli 2011	11	97	17	81	2.6%	0.48 [0.21, 1.10]	2011
Al-Jafari 2012	20	120	8	65	2.4%	1.43 [0.59, 3.44]	2012
Daoud 2013	41	226	11	103	3.1%	1.85 [0.91, 3.77]	2013
Abdel 2015	8	54	8	59	1.9%	1.11 [0.38, 3.19]	2015
Ahmadi 2015	57	115	14	89	3.3%	5.26 [2.67, 10.37]	2015
**Subtotal (95% CI)**		**2395**		**1803**	**53.2%**	**0.87 [0.62, 1.22]**	
Total events	421		347				
Heterogeneity: Tau² = 0.38; Chi² = 65.99, df = 17 (P < 0.00001); I² = 74%							
Test for overall effect: Z = 0.79 (P = 0.43)							
**Total (95% CI)**		**6959**		**9400**	**100.0%**	**0.87 [0.73, 1.03]**	
Total events	1291		1945				
Heterogeneity: Tau² = 0.12; Chi² = 80.19, df = 26 (P < 0.00001); I² = 68%							
Test for overall effect: Z = 1.65 (P = 0.10)							
Test for subgroup differences: Chi² = 0.00, df = 1 (P = 0.96), I² = 0%							

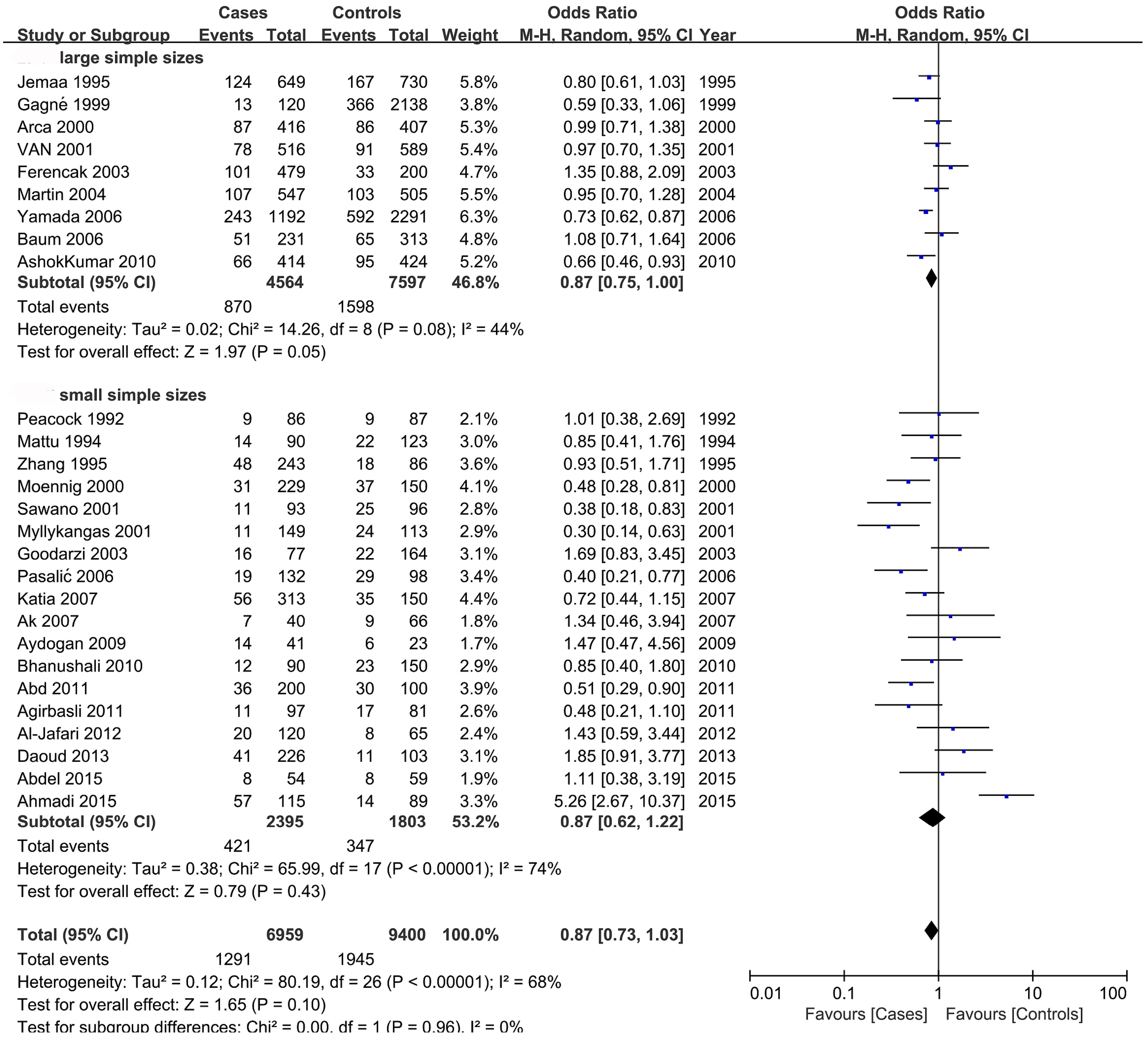

Supplementary Figure 4. Stratified analysis based on sample size for the association between the *LPL* S447X polymorphism and CAD risk using dominant genetic model (GG+GC vs. CC).

Study or Subgroup	Cases Events	Cases Total	Controls Events	Controls Total	Weight	Odds Ratio M-H, Random, 95% CI	Year
**large simple sizes**							
Arca 2000	17	416	17	407	15.2%	0.98 [0.49, 1.94]	2000
VAN 2001	34	631	14	606	17.5%	2.41 [1.28, 4.53]	2001
Martin 2004	13	547	12	505	11.7%	1.00 [0.45, 2.21]	2004
Izar 2009	74	379	76	589	43.2%	1.64 [1.15, 2.32]	2009
**Subtotal (95% CI)**		**1973**		**2107**	**87.6%**	**1.49 [1.03, 2.15]**	
Total events	138		119				
Heterogeneity: $Tau^2 = 0.05$; $Chi^2 = 4.86$, df = 3 (P = 0.18); $I^2 = 38\%$							
Test for overall effect: Z = 2.09 (P = 0.04)							
**small simple sizes**							
Zhang 1995	10	243	2	86	3.3%	1.80 [0.39, 8.40]	1995
Bhanushali 2010	1	90	4	150	1.6%	0.41 [0.05, 3.73]	2010
Rebhi 2012	203	212	101	104	4.5%	0.67 [0.18, 2.53]	2012
Abdel 2015	3	65	3	78	3.0%	1.21 [0.24, 6.21]	2015
**Subtotal (95% CI)**		**610**		**418**	**12.4%**	**0.94 [0.42, 2.10]**	
Total events	217		110				
Heterogeneity: $Tau^2 = 0.00$; $Chi^2 = 1.57$, df = 3 (P = 0.67); $I^2 = 0\%$							
Test for overall effect: Z = 0.14 (P = 0.89)							
**Total (95% CI)**		**2583**		**2525**	**100.0%**	**1.43 [1.07, 1.90]**	
Total events	355		229				
Heterogeneity: $Tau^2 = 0.02$; $Chi^2 = 7.72$, df = 7 (P = 0.36); $I^2 = 9\%$							
Test for overall effect: Z = 2.45 (P = 0.01)							
Test for subgroup differences: $Chi^2 = 1.02$, df = 1 (P = 0.31), $I^2 = 1.7\%$							

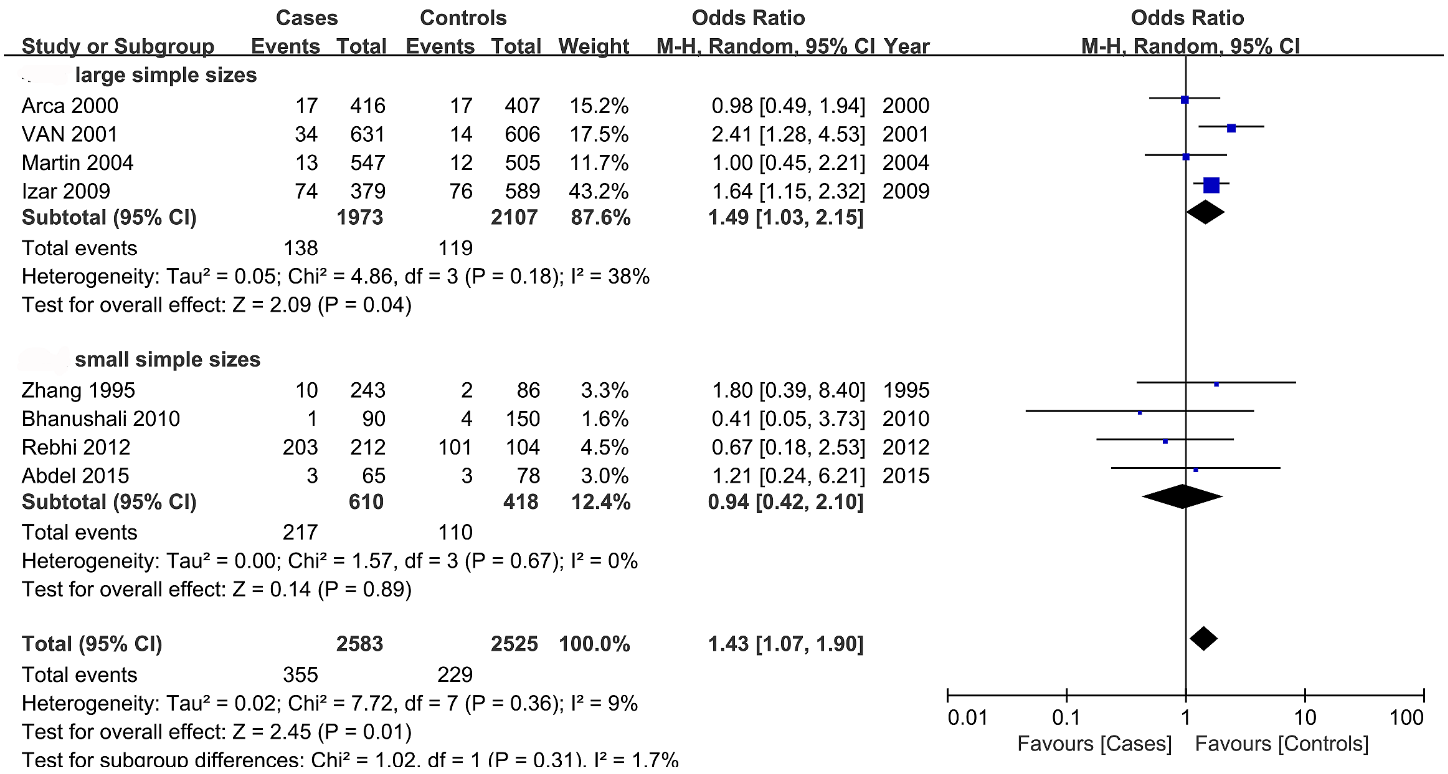

Supplementary Figure 5. Stratified analysis based on sample size for the association between the *LPL* D9N polymorphism and CAD risk using dominant genetic model (AA+GA vs. GG).

Study or Subgroup	Cases Events	Cases Total	Controls Events	Controls Total	Weight	Odds Ratio M-H, Fixed, 95% CI	Year
**large simple sizes**							
Wittrup 1997	101	1715	452	9214	35.7%	1.21 [0.97, 1.51]	1997
Arca 2000	18	416	16	407	4.1%	1.11 [0.56, 2.20]	2000
VAN 2001	20	599	22	664	5.4%	1.01 [0.54, 1.87]	2001
Ferencak 2003	7	479	8	200	3.0%	0.36 [0.13, 1.00]	2003
Keavney 2004	165	4524	116	3332	34.4%	1.05 [0.82, 1.34]	2004
Martin 2004	20	547	15	505	4.0%	1.24 [0.63, 2.45]	2004
Tripathi 2010	34	329	23	331	5.5%	1.54 [0.89, 2.68]	2010
**Subtotal (95% CI)**		**8609**		**14653**	**92.1%**	**1.13 [0.98, 1.30]**	
Total events	365		652				
Heterogeneity: Chi² = 7.03, df = 6 (P = 0.32); I² = 15%							
Test for overall effect: Z = 1.64 (P = 0.10)							
**small simple sizes**							
Moennig 2000	10	229	10	150	3.1%	0.64 [0.26, 1.58]	2000
Myllykangas 2001	9	149	3	113	0.9%	2.36 [0.62, 8.92]	2001
Rebhi 2012	1	212	1	104	0.4%	0.49 [0.03, 7.88]	2012
Abdel 2015	22	73	17	54	3.6%	0.94 [0.44, 2.01]	2015
**Subtotal (95% CI)**		**663**		**421**	**7.9%**	**0.96 [0.57, 1.60]**	
Total events	42		31				
Heterogeneity: Chi² = 2.76, df = 3 (P = 0.43); I² = 0%							
Test for overall effect: Z = 0.18 (P = 0.86)							
**Total (95% CI)**		**9272**		**15074**	**100.0%**	**1.11 [0.97, 1.28]**	
Total events	407		683				
Heterogeneity: Chi² = 10.27, df = 10 (P = 0.42); I² = 3%							
Test for overall effect: Z = 1.53 (P = 0.13)							
Test for subgroup differences: Chi² = 0.38, df = 1 (P = 0.54), I² = 0%							

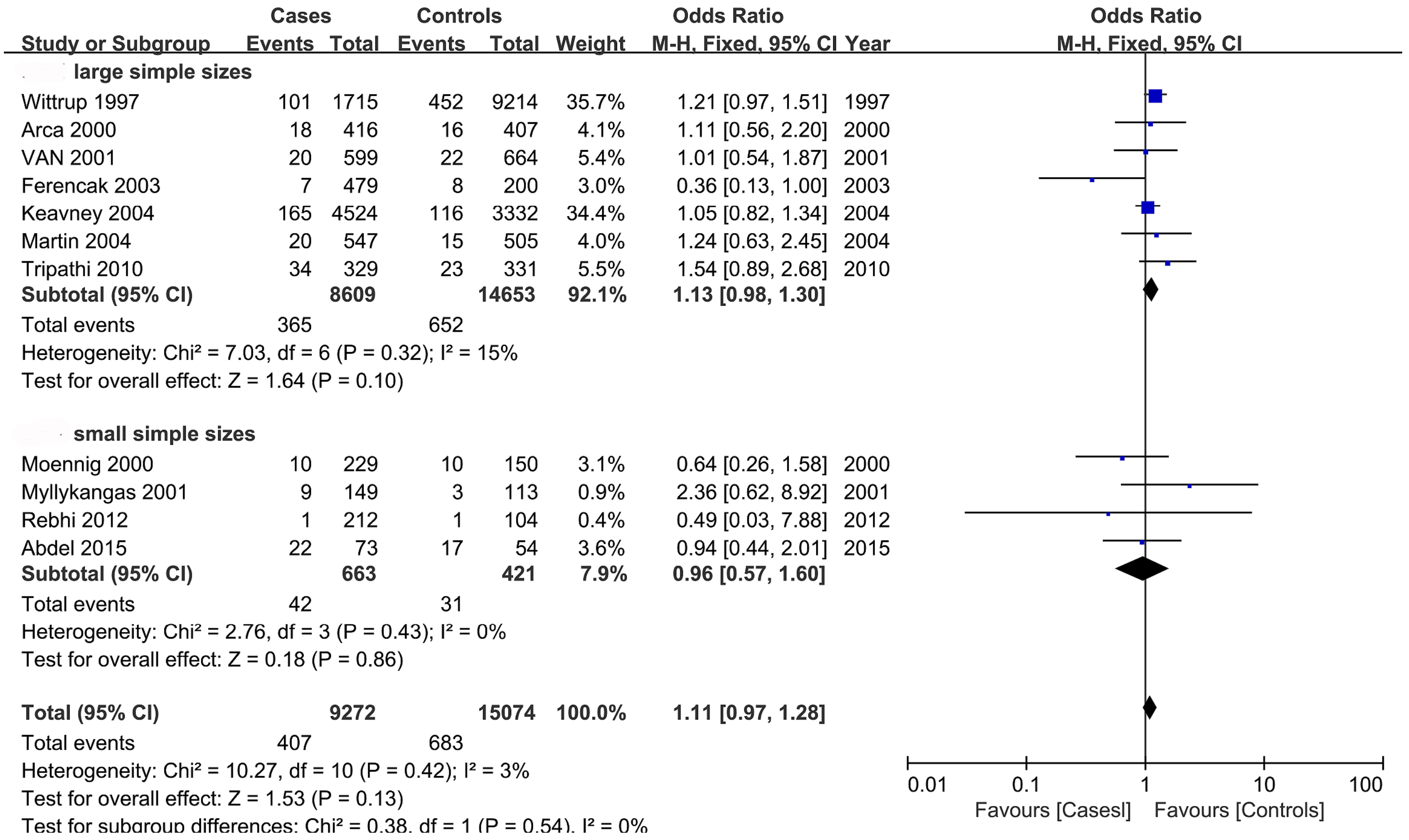

Supplementary Figure 6. Stratified analysis based on sample size for the association between the *LPL* N291S polymorphism and CAD risk using dominant genetic model (GG+GA vs. AA).

Study or Subgroup	Cases Events	Cases Total	Controls Events	Controls Total	Weight	Odds Ratio M-H, Fixed, 95% CI	Year
**Caucasians**							
Thorn 1990	45	60	63	93	1.0%	1.43 [0.69, 2.96]	1990
Peacock 1992	56	85	61	90	1.7%	0.92 [0.49, 1.72]	1992
Mattu 1994	62	90	87	123	1.9%	0.92 [0.51, 1.66]	1994
Jemaa 1995	430	614	544	732	12.4%	0.81 [0.64, 1.03]	1995
Wang 1996	247	350	87	125	3.2%	1.05 [0.67, 1.63]	1996
Stepanov 1998	67	93	90	119	1.8%	0.83 [0.45, 1.54]	1998
Anderson 1999	341	483	108	168	3.9%	1.33 [0.92, 1.93]	1999
Isbir 2003	63	100	52	72	1.9%	0.65 [0.34, 1.26]	2003
Keavney 2004	3612	4569	2656	3377	53.5%	1.02 [0.92, 1.14]	2004
Duman 2004	53	78	35	49	1.2%	0.85 [0.39, 1.85]	2004
Georgiev 2008	84	109	27	32	0.8%	0.62 [0.22, 1.78]	2008
**Subtotal (95% CI)**		**6631**		**4980**	**83.4%**	**0.99 [0.91, 1.08]**	
Total events	5060		3810				
Heterogeneity: Chi² = 9.53, df = 10 (P = 0.48); I² = 0%							
Test for overall effect: Z = 0.24 (P = 0.81)							
**Asians**							
Abu-Amero 2003	293	431	329	511	8.1%	1.17 [0.90, 1.54]	2003
Al-Jafari 2012	70	120	40	65	1.8%	0.88 [0.47, 1.62]	2012
Daoud 2013	137	226	57	103	2.6%	1.24 [0.78, 1.99]	2013
Bahrami 2015	133	211	131	203	4.1%	0.94 [0.63, 1.40]	2015
**Subtotal (95% CI)**		**988**		**882**	**16.6%**	**1.09 [0.90, 1.33]**	
Total events	633		557				
Heterogeneity: Chi² = 1.62, df = 3 (P = 0.66); I² = 0%							
Test for overall effect: Z = 0.91 (P = 0.36)							
**Total (95% CI)**		**7619**		**5862**	**100.0%**	**1.01 [0.93, 1.09]**	
Total events	5693		4367				
Heterogeneity: Chi² = 11.99, df = 14 (P = 0.61); I² = 0%							
Test for overall effect: Z = 0.16 (P = 0.88)							
Test for subgroup differences: Chi² = 0.86, df = 1 (P = 0.35), I² = 0%							

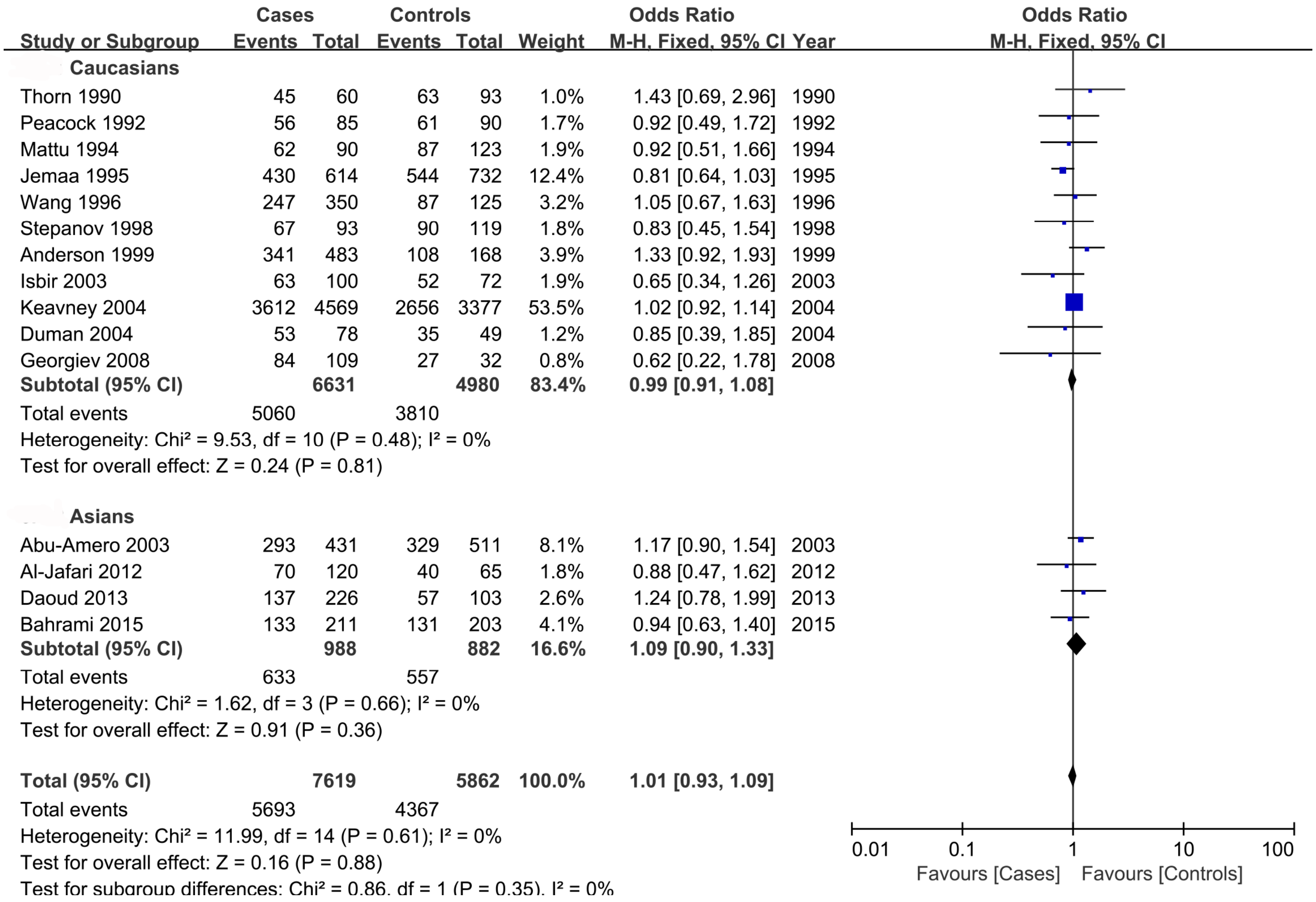

Supplementary Figure 7. Stratified analysis based on ethnicity for the association between the *LPL* PvuII polymorphism and CAD risk using dominant genetic model (TT+CT vs. CC).

Study or Subgroup	Cases Events	Cases Total	Controls Events	Controls Total	Weight	Odds Ratio M-H, Random, 95% CI	Year
**large simple sizes**							
Jemaa 1995	430	614	544	732	11.0%	0.81 [0.64, 1.03]	1995
Anderson 1999	341	483	108	168	4.6%	1.33 [0.92, 1.93]	1999
Abu-Amero 2003	293	431	329	511	8.6%	1.17 [0.90, 1.54]	2003
Keavney 2004	3612	4569	2656	3377	52.8%	1.02 [0.92, 1.14]	2004
**Subtotal (95% CI)**		**6097**		**4788**	**77.0%**	**1.03 [0.87, 1.23]**	
Total events	4676		3637				
Heterogeneity: Tau² = 0.02; Chi² = 6.70, df = 3 (P = 0.08); I² = 55%							
Test for overall effect: Z = 0.39 (P = 0.70)							
**small simple sizes**							
Thorn 1990	45	60	63	93	1.2%	1.43 [0.69, 2.96]	1990
Peacock 1992	56	85	61	90	1.6%	0.92 [0.49, 1.72]	1992
Mattu 1994	62	90	87	123	1.8%	0.92 [0.51, 1.66]	1994
Wang 1996	247	350	87	125	3.2%	1.05 [0.67, 1.63]	1996
Stepanov 1998	67	93	90	119	1.7%	0.83 [0.45, 1.54]	1998
Isbir 2003	63	100	52	72	1.5%	0.65 [0.34, 1.26]	2003
Duman 2004	53	78	35	49	1.0%	0.85 [0.39, 1.85]	2004
Georgiev 2008	84	109	27	32	0.6%	0.62 [0.22, 1.78]	2008
Rebhi 2012	152	212	84	104	1.9%	0.60 [0.34, 1.07]	2012
Al-Jafari 2012	70	120	40	65	1.7%	0.88 [0.47, 1.62]	2012
Daoud 2013	137	226	57	103	2.9%	1.24 [0.78, 1.99]	2013
Bahrami 2015	133	211	131	203	3.9%	0.94 [0.63, 1.40]	2015
**Subtotal (95% CI)**		**1734**		**1178**	**23.0%**	**0.92 [0.78, 1.09]**	
Total events	1169		814				
Heterogeneity: Tau² = 0.00; Chi² = 7.12, df = 11 (P = 0.79); I² = 0%							
Test for overall effect: Z = 0.99 (P = 0.32)							
**Total (95% CI)**		**7831**		**5966**	**100.0%**	**1.00 [0.92, 1.08]**	
Total events	5845		4451				
Heterogeneity: Tau² = 0.00; Chi² = 15.02, df = 15 (P = 0.45); I² = 0%							
Test for overall effect: Z = 0.08 (P = 0.94)							
Test for subgroup differences: Chi² = 0.95, df = 1 (P = 0.33), I² = 0%							

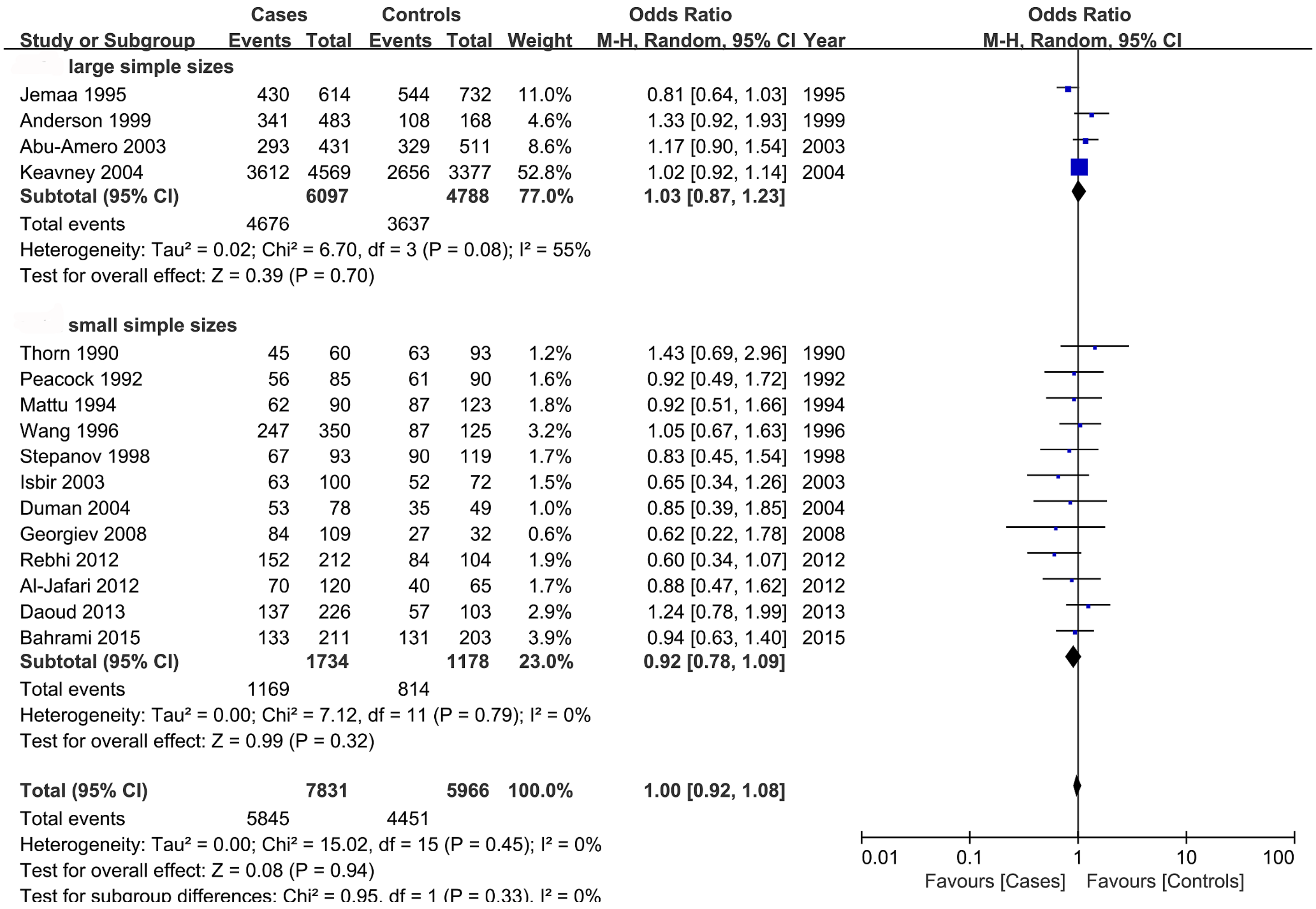

Supplementary Figure 8. Stratified analysis based on sample size for the association between the *LPL* PvuII polymorphism and CAD risk using dominant genetic model (TT+CT vs. CC).

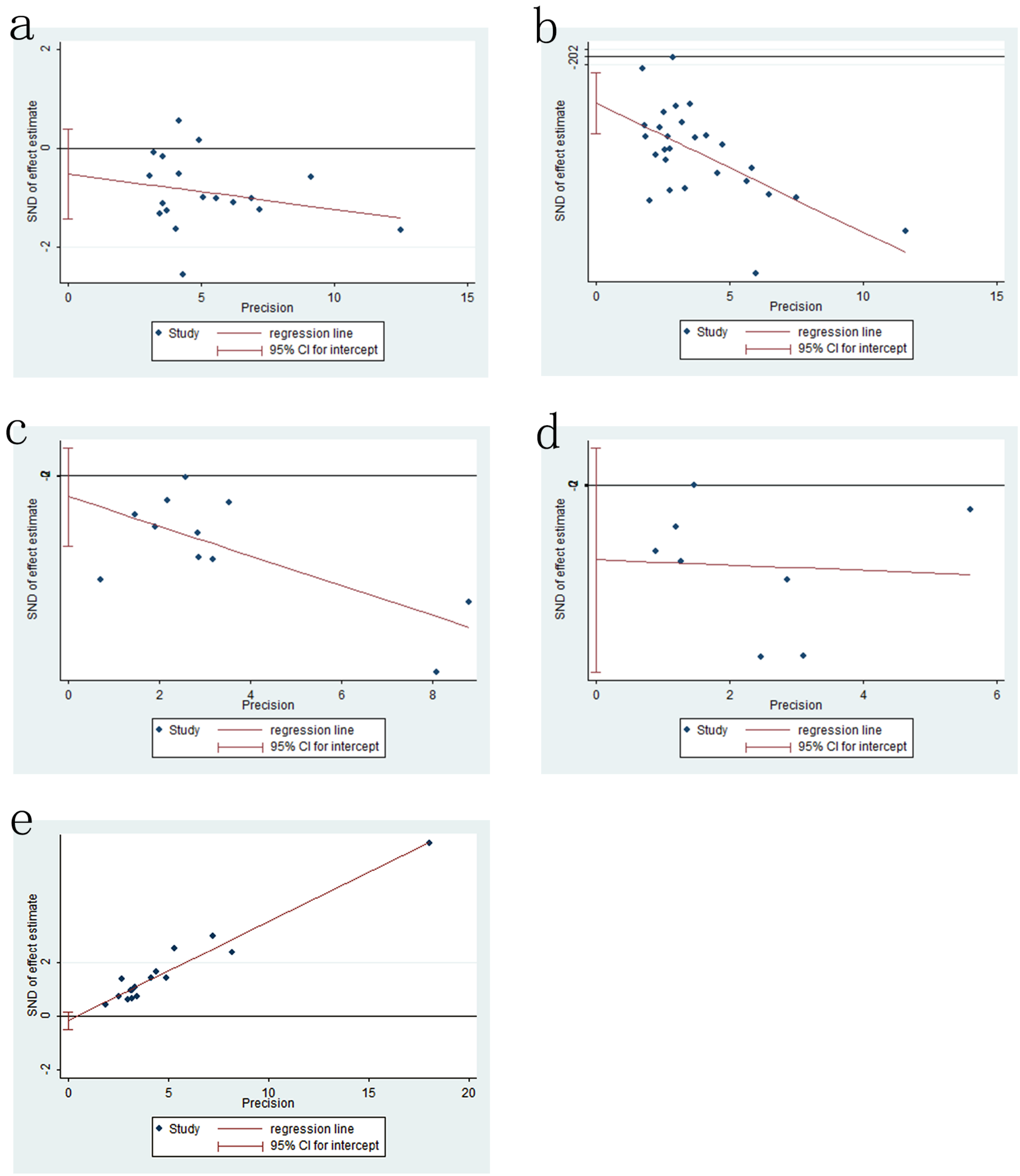

Supplementary Figure 9. Egger's regression test of publication bias for the association between the *LPL* gene polymorphisms and susceptibility to CAD. (a). HindIII polymorphism; (b). S447X polymorphism; (c). N291S polymorphism; (d). D9N polymorphism; (e). PvuII polymorphism.

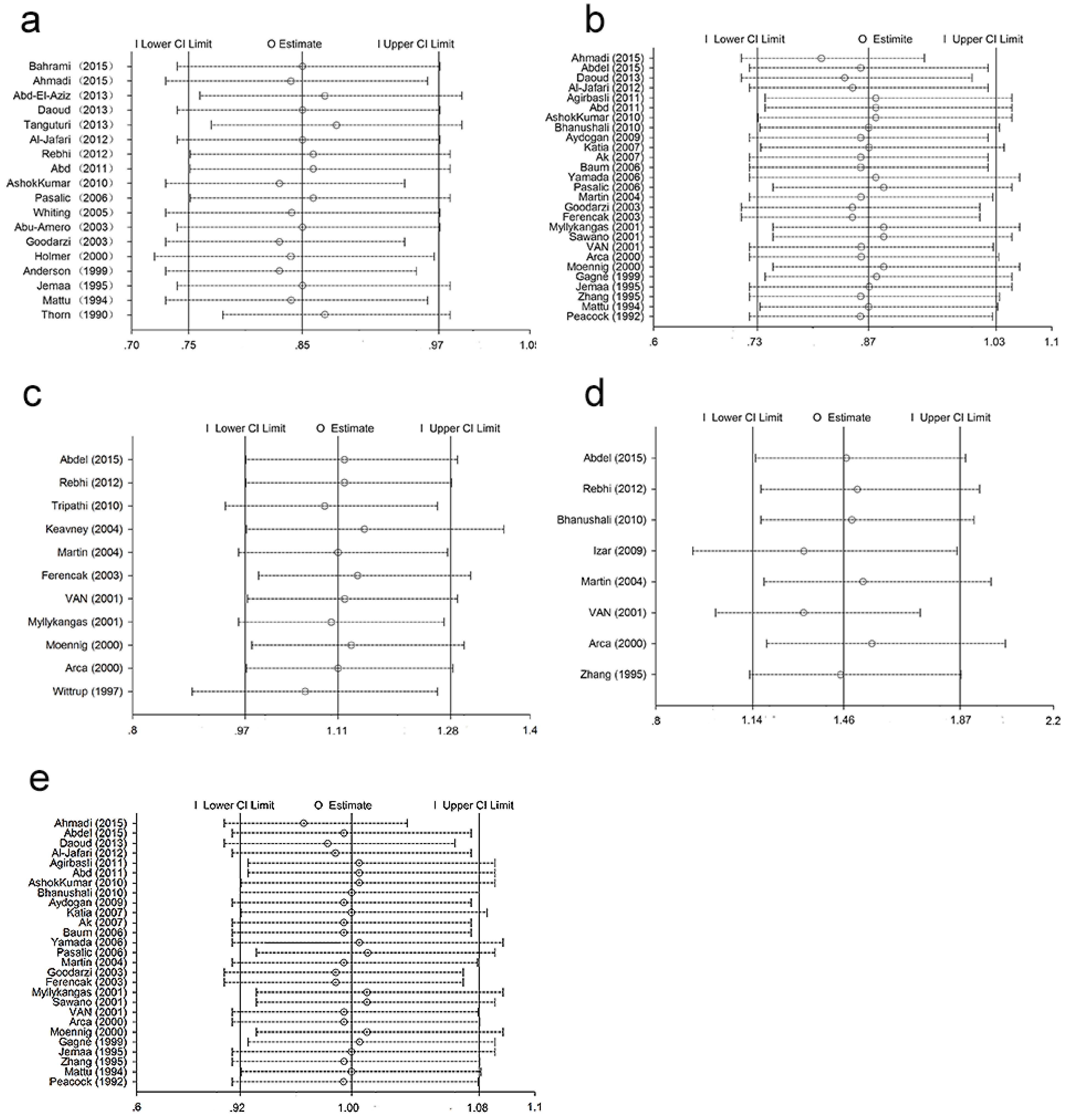

Supplementary Figure 10. Sensitivity analysis on the correlation between *LPL* gene polymorphisms and susceptibility to CAD. (a). sensitivity analysis for HindIII and CAD risk; (b). sensitivity analysis for S447X and CAD risk; (c). sensitivity analysis for N291S and CAD risk; (d). Sensitivity analysis for D9N and CAD risk; (e). sensitivity analysis for PvuII and CAD risk;
